# The efficacy and safety of thrombopoietin receptor agonists in solid tumors with chemotherapy-induced thrombocytopenia: a systematic review and network meta-analysis of randomized controlled trials

**DOI:** 10.3389/fphar.2025.1683857

**Published:** 2025-12-01

**Authors:** Yingyu Lai, Qianni Pan, Shiyu Wang, Qingmao Luo, Zhencong Huang, Mingzhong Wei, Zhouqian Jiang, Wenyan Yi

**Affiliations:** Department of Pharmacy, The People’s Hospital of Hezhou, Hezhou, China

**Keywords:** thrombopoietin receptor agonists, solid tumors, chemotherapy-induced thrombocytopenia, network meta-analysis, avatrombopag, eltrombopag, romiplostim, hetrombopag

## Abstract

**Objective:**

The objective of this study was to compare and rank the efficacy and safety of different thrombopoietin receptor agonists (TPO-RAs) in the treatment of chemotherapy-induced thrombocytopenia (CIT) among patients with solid tumors.

**Methods:**

PubMed, Cochrane Library, Embase, MEDLINE, Web of Science, ClinicalTrials.gov, CNKI, Wanfang Database, VIP Database, SinoMed, and China Drug Trials (www.chinadrugtrials.org.cn) were searched for randomized controlled trials (RCTs) of TPO-RAs for CIT in solid tumors from the inception to 31 December 2024. The Cochrane Risk of Bias Assessment Tool 2 was used for assessing the risk of bias. We performed a random-effects network meta-analysis using STATA 14.0 software. Treatments were ranked according to the surface under the cumulative ranking curve. Confidence of the evidence was assessed using Confidence in Network Meta-Analysis. The study protocol was registered with PROSPERO (number CRD42024612536).

**Results:**

A total of eight studies (568 patients) were included. Most RCTs (7/8) showed a low risk of bias. The confidence in evidence was often low or very low. Our network meta-analysis indicates that when compared with placebo, hetrombopag (summary RR 0.45, 95% confidence interval 0.28–0.73) and eltrombopag (0.57, 0.41–0.81) significantly reduced the incidence of chemotherapy dose reduction or delay due to thrombocytopenia. Hetrombopag (0.29, 0.13–0.68) also significantly reduced the platelet transfusions. Eltrombopag had the lowest risk for bleeding event (0.41, 0.13–1.23) and mortality (0.83, 0.48–1.44). There were no significant differences in the risk of adverse events (AEs) between interventions. Hetrombopag (0.37, 0.02–8.68) showed the least risk of thrombosis. According to rankograms, hetrombopag was ranked as the best for reducing the incidence of chemotherapy dose reduction or delay, and platelet transfusions, with the least risk of serious AEs and thrombosis. Eltrombopag carried the least risk of bleeding events and mortality.

**Conclusion:**

Our network meta-analysis suggested that based on the limited indirect data, hetrombopag may represent the preferred therapy for avoiding chemotherapy dose reductions or delays and platelet transfusion. Eltrombopag may be considered the preferred therapeutic option for avoiding bleeding events and mortality. Both compounds have acceptable safety profiles. However, larger head-to-head trials are needed to confirm these findings.

**Systematic Review Registration:**

https://www.crd.york.ac.uk/PROSPERO/view/CRD42024612536, identfier CRD42024612536.

## Introduction

With the aging of the population, the increasing number of cancer patients imposes a significant burden on the healthcare system (Alwahsh et al., 2024). Chemotherapy-induced thrombocytopenia (CIT), a common adverse reaction to antineoplastic therapy, refers to the inhibitory effect of antineoplastic therapy on bone marrow, especially on megakaryocytic cells, resulting in a lower platelet count in peripheral blood than the normal value (typically <100 × 10^9^/L). CIT occurred in approximately 10%–40% of patients with solid tumors (Griffiths et al., 2022; Goldberg et al., 1994). Although CIT has historically only occurred in cytotoxic and myelosuppressive chemotherapy, antineoplastic therapy now also include many targeted therapies and immune checkpoint inhibitors (ICIs), which can also result in CIT due to alternative mechanisms (Kuter, 2022). There have been quite a few reports on thrombocytopenia caused by ICIs and cellular immunotherapy. Although the incidence of ICIs-induced thrombocytopenia is lower, some patients may experience persistent and recurrent severe thrombocytopenia and bleeding, which can even be life-threatening in severe cases (Wang et al., 2019). Many factors related to the patient (such as the age, type of tumor, number of previous chemotherapy cycles, and extent of bone marrow tumor involvement) determine the severity of CIT (Kuter, 2022). Chemotherapy regimens that include gemcitabine or platinum agents usually carry the highest risk of CIT (Shaw et al., 2021). CIT is defined as a platelet count below 100 × 10^9^/L as a direct consequence of myelosuppressive chemotherapy. According to the National Cancer Institute Common Terminology Criteria for Adverse Events version 5.0, the severity of thrombocytopenia is graded as follows: grade 1: platelet count < lower limit of normal to 75 × 10^9^/L, grade 2: platelet count 75 to 50 × 10^9^/L, grade 3: platelet count 50 to 25 × 10^9^/L, grade 4: platelet count <25 × 10^9^/L, and grade 5: death. When the platelet count falls below 50 × 10^9^/L, mucocutaneous bleeding may occur, and the patient is at increased risk during surgery or traumatic examinations. When the platelet count falls below 20 × 10^9^/L, there is a high risk of spontaneous bleeding. When it is less than 10 × 10^9^/L, there is a very high risk of spontaneous bleeding, and platelet transfusion is requisite to prevent major bleeding (Kuter, 2022). CIT not only increases the risk of bleeding but can also lead to chemotherapy dose reduction or delay, treatment discontinuation, higher treatment costs, reduced treatment efficacy, poor prognosis, and even death. There is an urgent need for safe and effective therapies for CIT. Currently, treatment options include platelet transfusion and the use of thrombopoiesis-promoting drugs. The need for platelet transfusion in CIT is rather uncommon due to scarce resources, temporary improvement, and the additional risk of infusion-related complications and immuno-suppressive effects (Schmied et al., 2021; Chen et al., 2023). Except in patients with platelet counts below 25 × 10^9^/L with significantly increased bleeding rates, platelet transfusion is the only treatment (Kuter, 2022). Nonetheless, platelet counts below 100 × 10^9^/L present a challenge. The thrombopoiesis-promoting drugs primarily consist of traditional platelet-boosting agents such as recombinant human interleukin-11 (rhIL-11), recombinant human thrombopoietin (rhTPO), and novel thrombopoietin receptor agonists (TPO-RAs). Despite extensive research over several decades into thrombopoiesis-promoting drugs for CIT, no agents have currently been approved by the U.S. FDA or EMA for CIT (Al-Samkari, 2024). Recombinant interleukin 11 (oprelvekin, Neumega®) demonstrated a reduction in the need for platelet transfusions from 96% to 70% among patients who had previously received platelet transfusions in an earlier chemotherapy cycle and subsequently underwent further chemotherapy. However, it is associated with significant adverse effects, such as arrhythmias, fluid retention, and pulmonary edema (Tepler et al., 1996). First-generation thrombopoietic agents including rhTPO and recombinant human megakaryocyte growth and development factor (PEG-rHuMGDF) have demonstrated efficacy in raising platelet counts in a variety of clinical settings (Soff et al., 2022). Around 2000, the development of both agents in the West was discontinued due to concerns about the formation of antidrug antibodies that cross-reacted with endogenous TPO, causing thrombocytopenia (Chen et al., 2023; Li et al., 2001; Soff et al., 2019). The development of rhTPO (TPIAO®) persisted and was completed in China, where it has subsequently been licensed for the treatment of CIT (https://www.mims.com/thailand/drug/info/tpiao) and where its use is recommended by Chinese oncological practice guidelines (Consensus Committee Of Chemotherapy Induced Thrombocytopenia, 2018). In recent years, TPO-RA drugs including hetrombopag, eltrombopag, avatrombopag, and romiplostim have demonstrated favorable efficacy in increasing the platelet levels. However, the National Comprehensive Cancer Network (NCCN) Guidelines: Hematopoietic Growth Factors version 1.2025 (Griffiths et al., 2024) points out that insufficient data are available to support the use of TPO-RAs other than romiplostim for CIT outside of a clinical trial. Few studies compare the efficacy and safety of these TPO-RAs for CIT (Song and Al-Samkari, 2023). Therefore, we performed a network meta-analysis to compare the efficacy and safety profiles of different TPO-RAs for CIT among patients with solid tumors to provide more evidence for clinical decision-making.

## Methods

We carried out a systematic review and network meta-analysis of randomized controlled trials (RCTs) according to PRISMA guidelines (Hutton et al., 2015).The study protocol was registered with PROSPERO (number CRD42024612536).

### Identification of studies

PubMed, Cochrane Library, Embase, MEDLINE, Web of Science, ClinicalTrials.gov, CNKI, Wanfang Database, VIP Database, SinoMed, and China Drug Trials (www.chinadrugtrials.org.cn) were systematically searched to identify potentially eligible studies. All documents were searched from the beginning of the database until 31 December 2024, without restrictions of country or language. The search keywords were “romiplostim, eltrombopag, avatrombopag, hetrombopag, lusutrombopag, neoplasms, and thrombocytopenia.” All keywords were searched in the Title/Abstract. We included RCTs of TPO-RAs for the treatment of CIT in adult patients with solid tumors. Detailed search terms are listed in Supplementary Material Appendix 1 search strategy.

### Eligibility criteria

The eligibility criteria were as follows: (1) research design: RCTs comprising any of the following interventions: avatrombopag, lusutrombopag, eltrombopag, romiplostim, and hetrombopag. (2) Population: patients older than 18 years with solid tumors treated with chemotherapy, not limited to specific types of solid tumors. No limits were imposed on the gender and age of patients. (3) Comparator(s) or control(s): the control arm consisted of placebo or no treatment or one of the above interventions. The following studies were excluded: duplicated literature, studies including incomplete or incorrect data, reviews and systematic reviews, nonhuman studies, case reports, observational research, cohort studies, retrospective analysis, pharmacokinetics, and articles unrelated to the topic of this study.

### Data extraction

Two reviewers (LYY and PQN) independently performed the literature search, screened the search results, retrieved full-text articles, and checked the eligibility criteria. Differences were discussed, and a third reviewer (LQM) was contacted if consensus was not reached. Two reviewers independently extracted data from all the selected trials on electronic forms. Disagreements between reviewers were resolved by discussion or decided by the third party. Study authors were contacted in case of missing or unclear information. The data included in the extraction table were as follows: (1) author and years, regions and registration number of trails, random and blinding method, and total number of participants; (2) age and gender of the patients; (3) intervention characteristics (type, dose, and duration); (4) outcome indicators: bleeding events, platelet transfusion, chemotherapy dose reduction or delays, incidence of grade 3 or 4 thrombocytopenia, and incidence of platelet count >400 × 10^9^/L; (5) safety data: adverse events (AEs), serious AEs, thrombosis, and mortality.

### Risk of bias assessment

The Cochrane Risk of Bias Assessment tool (RoB 2.0) (Sterne et al., 2019) was used for assessing the risk of bias. Potential sources of bias include the randomization process, deviations from the intended interventions, missing outcome data, measurement of the outcome, and selection of the reported result. Each trial received a study level score of low, high, or some concerns for each domain. The overall risk of bias was classified into high, some concerns, or low. Two reviewers (LYY and WSY) independently conducted this assessment, and discrepancies were resolved by consensus. If consensus was not reached, a third reviewer (LQM) was contacted.

### Outcomes

The efficacy outcomes of our systematic review included chemotherapy dose reduction or delay due to thrombocytopenia, platelet transfusions, incidence of grade 3 or 4 thrombocytopenia, and bleeding events. The safety outcomes were as follows: platelet count >400 × 10^9^/L, AEs, serious AEs, thrombosis, and mortality.

### Data synthesis

We performed frequentist network meta-analyses using the “network” packages in Stata 14.0 software (StataCorp, College Station, TX, United States). All outcomes were binary outcomes. In addition, the effect size was the risk ratio (RR). Network plots were drawn for each outcome to visualize the network geometry and node connectivity (Chaimani et al., 2013). The diagrams were designed so that the size of the nodes represents the sample size of the intervention. Treatments with direct comparisons are linked with a line, and its thickness corresponds to the number of studies comparing each intervention. Global consistency and node-splitting methods were utilized to test for inconsistencies in the study results, with a p-value of <0.05 indicating inconsistency (Cui et al., 2025). I^2^ statistics and chi-square tests were used to assess heterogeneity. Data were combined using a random-effects model within a frequentist framework. We assumed network consistency and a common heterogeneity parameter across all treatment contrasts. For all treatment comparisons, we present the summary RR and 95% confidence intervals that account for uncertainty in variance estimates (Jackson and Riley, 2014) in league tables. To generate the surface under the cumulative ranking curve (SUCRA) values and obtain treatment hierarchies, we used a parametric bootstrap procedure with 5,000 resamples to compute ranking probabilities (White et al., 2012). To graphically present the distribution of ranking probabilities, the plots of ranking probabilities (rankograms) were drawn (Salanti et al., 2011).

### Certainty of evidence

The certainty of evidence produced by the synthesis for each outcome was evaluated using the framework described by Salanti et al. (2014) and implemented using the CINeMA (Confidence in Network Meta-Analysis) web application, which allows confidence in the results to be graded as high, moderate, low, and very low (Papakonstantinou et al., 2020). Six domains that affect the level of confidence in the network meta-analysis results are considered: (a) within-study bias, (b) reporting bias, (c) indirectness, (d) imprecision, (e) heterogeneity, and (f) incoherence (Papakonstantinou et al., 2020). For the primary outcome, we examined the confidence of evidence of all comparisons.

## Results

### Study characteristics

**FIGURE 1:**
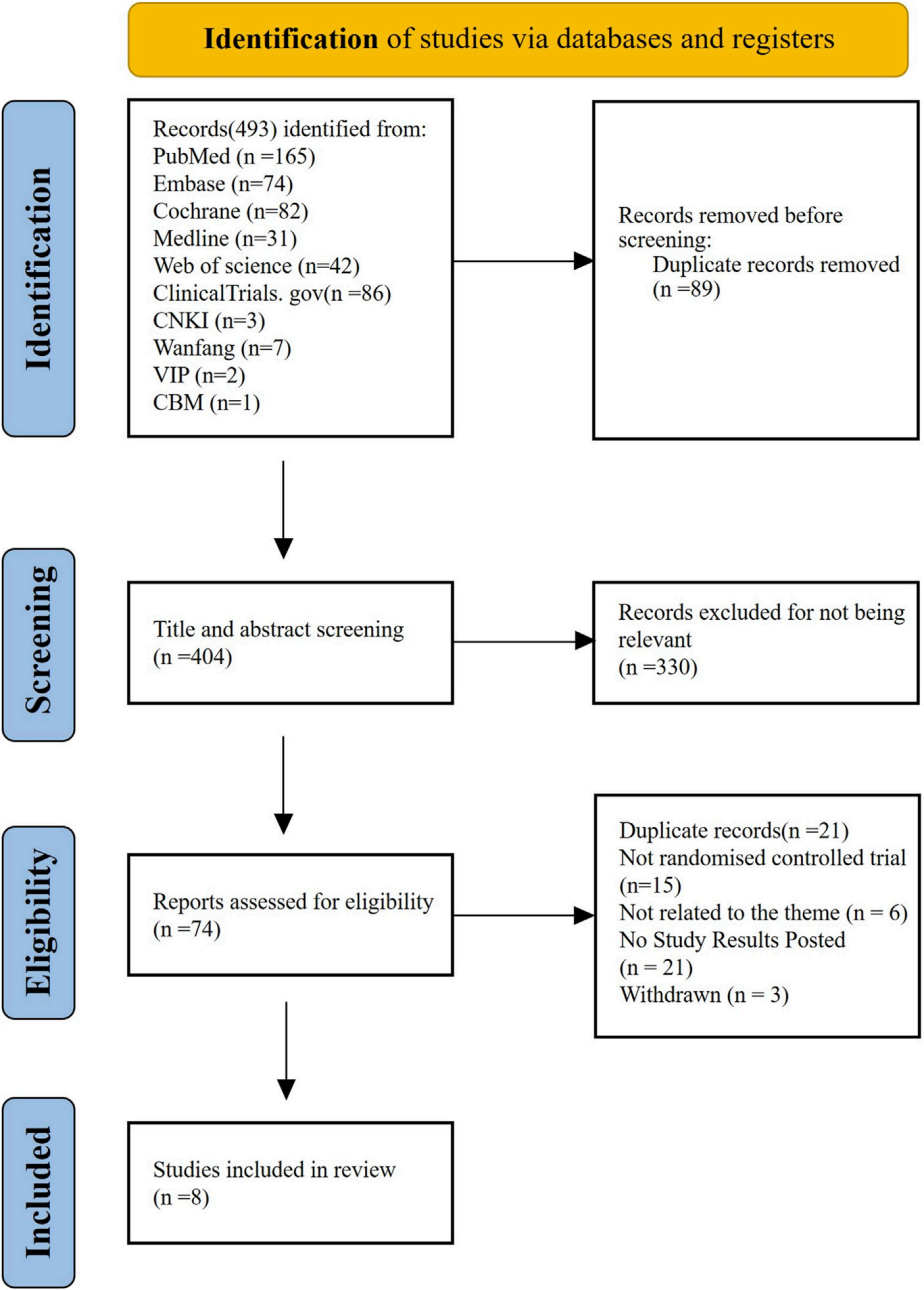
PRISMA flow diagram of the screening and selection process used in the study.

Our search identified 493 citations, of which 404 citations remained after removing duplicates. After screening 404 titles and abstracts and 74 full texts, eight RCTs (EUCTR, 2014; Qin et al., 2024; Natale et al., 2009; Kellum et al., 2010; Winer et al., 2015; Winer et al., 2017; Al-Samkari et al., 2022; Soff et al., 2019) with data for 568 participants met our inclusion criteria (Figure 1). The mean age ranged from 50 years to 69 years (for trials that reported the mean or median age). Sixty-five percent (n = 258) were women. Four interventional arms were included as follows: three studies with eltrombopag (Kellum et al., 2010; Winer et al., 2015; Winer et al., 2017), three studies with romiplostim (Soff et al., 2019; Natale et al., 2009; EUCTR, 2014), one with avatrombopag (Al-Samkari et al., 2022), and one with hetrombopag (Qin et al., 2024). Placebo was used as the control in all RCTs except in one study (Soff et al., 2019), where observation was used as the control. The trials were conducted in multiple countries from 2009 to 2024. Table 1 lists the characteristics of the included studies.

**TABLE 1:** Characteristics of the included studies evaluating TPO-RAs for CIT.

Study ID	Regions	Registration number	Stage	Randomization and blinding method	Sponsor	Chemotherapy	Interventions	Dose	Participants	Age (years)	Gender (M/F)
Kellum et al. (2010)	USA, European Union, Asia, and South America	NCT00102726	Phase 2	1:1:1:1 randomized, double-blind	GlaxoSmithKline	Carboplatin/paclitaxel	Eltrombopag	50 mg	44	58.5 (35–75)	23/21
							Eltrombopag	75 mg	44	59 (33–75)	16/28
							Eltrombopag	100 mg	46	58 (34–81)	24/22
							Placebo		46	58 (23–73)	16/30
Winer et al. (2015)	United States, Europe, and India	NCT01147809	Phase 1	3:1 randomized, double-blind	GlaxoSmithKline	Gemcitabine plus platinum	Eltrombopag	100 mg	9	53 (34–75)	2/7
							Placebo		3	55 (49–56)	2/1
						Gemcitabine monotherapy	Eltrombopag	100 mg	10	69 (50–74)	7/3
							Placebo		4	67.5 (31–81)	1/3
Winer et al. (2017)	United States, Europe, and India	NCT01147809	Phase 2	2:1 randomized, double-blind	GlaxoSmithKline	Gemcitabine plus platinum	Eltrombopag	100 mg	22	67.0 (51–80)	12/10
							Placebo		11	64.0 (57–83)	5/6
						Gemcitabine monotherapy	Eltrombopag	100 mg	30	67.5 (36–82)	17/13
							Placebo		12	66.0 (44–79)	5/7
Al-Samkari et al. (2022)	China, Hungary, Poland, and Russia	NCT03471078	Phase 3	2:1 randomized, double-blind	Sobi, Inc	Gemcitabine and fluorouracil	Avatrombopag	60 mg	82	61.0 (38–78)	39/43
							Placebo		40	60.8 (26–77)	18/22
Soff et al. (2019)	United States	NCT02052882	Phase 2	2:1 randomized, open label	Memorial Sloan Kettering cancer center	Carboplatin or cisplatin	Romiplostim	2.0 mg/kg/w, then titrated	15	50 (30–76)	5/10
							Observation		8	67 (46–77)	6/2
Natale et al. (2009)	USA	NCT00413283	Phase 2	4:1 randomized, double-blind	Amgen	Gemcitabine plus platinum	Romiplostim	250 µg	16	63.8 ± 10.8	12/4
							Romiplostim	500 µg	18	62.5 ± 7.7	12/6
							Romiplostim	750 µg	17	65.4 ± 8.2	15/2
							Placebo		12	59.8 ± 6.6	6/6
EUCTR (2014)	Germany	NCT03622931	Phase 2	1:1randomized, double-blind	GMIHO Gesellschaft für Medizinische Innovation - Hämatologie und Onkologie mbH	Myelosuppressive chemotherapy	Romiplostim	750 µg	11	18–64 years:6 65–84 years: 5	0/11
							Placebo		10	18–64 years:7 65–84 years: 3	0/10
Qin et al. (2024)	China	NCT03976882	Phase 2	1:1randomized	Jiangsu HengRui medicine co., Ltd	Gemcitabine and carboplatin	Hetrombopag	7.5 mg	28	58 (38–74)	13/15
							Placebo		31	58 (36–72)	18/13

### Risk of bias in the included studies

**FIGURE 2:**
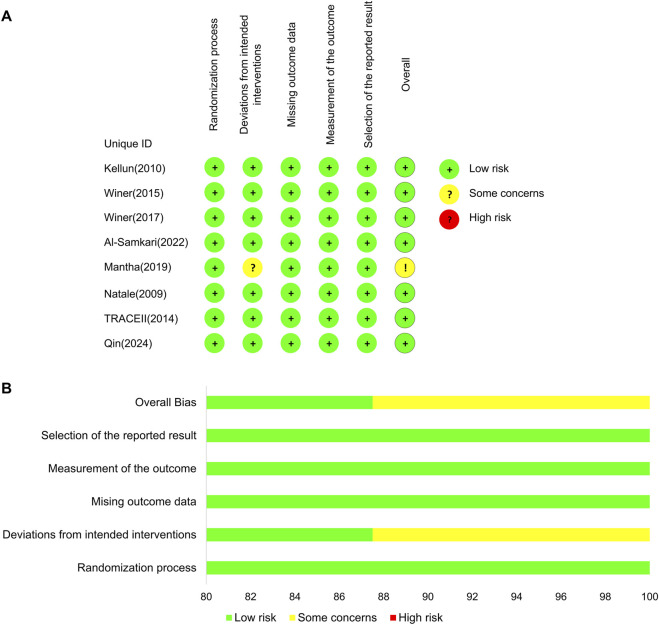
Bias risk assessment results. **(A)** Risk-of-bias assessment for the individual studies. **(B)** Risk-of-bias assessment for the individual domains.

The results of the risk-of-bias assessment are shown in Figure 2. Most RCTs showed a low risk of bias because their protocols and outcomes were well described in each study, except one of the studies that showed some concerns in the domain of deviations from intended interventions (Soff et al., 2019).

### Outcomes

Chemotherapy dose reduction or delay due to thrombocytopenia

**FIGURE 3:**
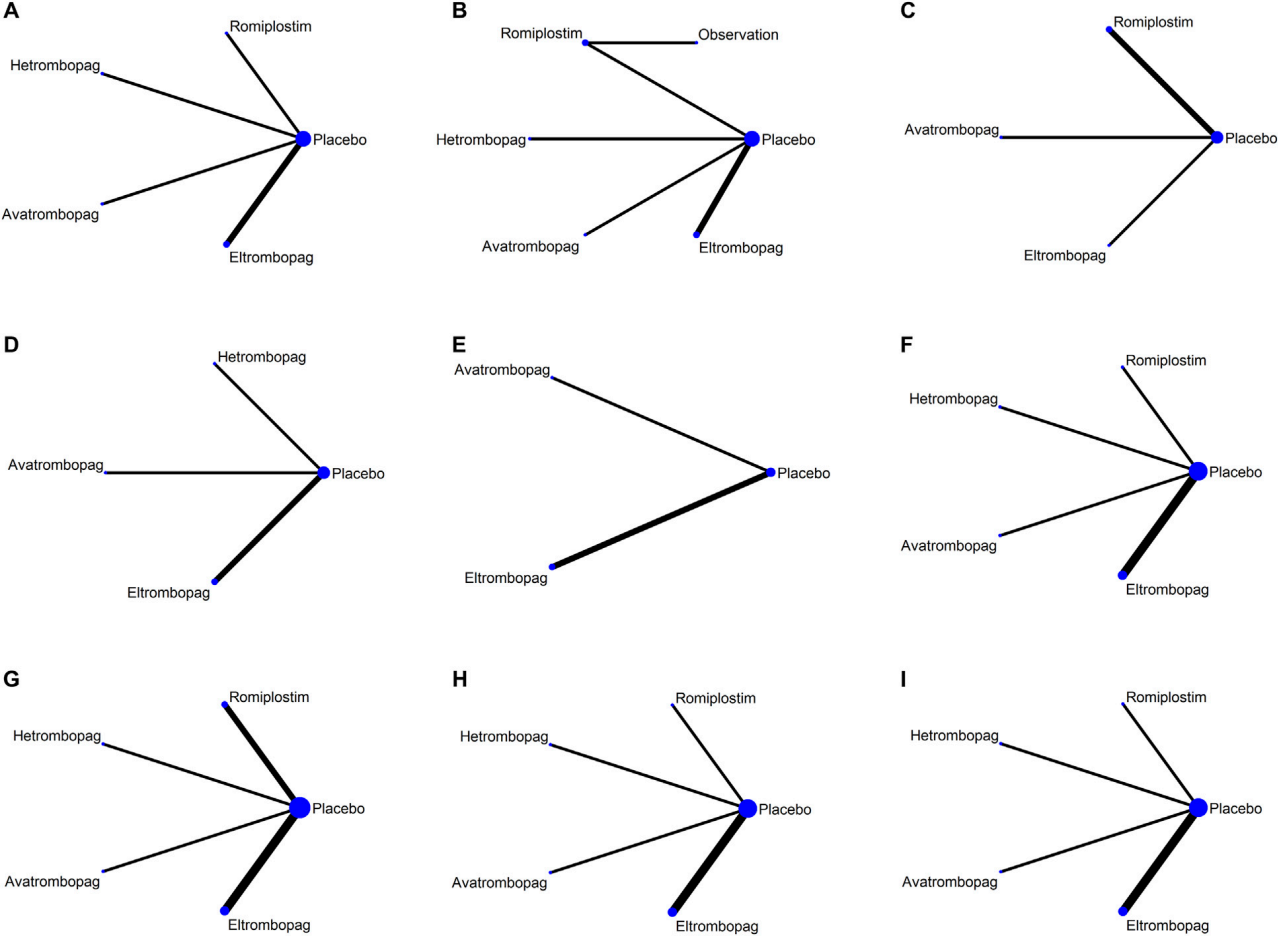
Network map for all outcomes. **(A)** Chemotherapy dose reduction or delay due to thrombocytopenia, **(B)** platelet transfusions, **(C)** incidence of grade 3 or 4 thrombocytopenia, **(D)** bleeding events, **(E)** platelet count >400 × 109/L, **(F)** AES, **(G)** serious AES, **(H)** thrombosis, and **(I)** mortality.

**FIGURE 4:**
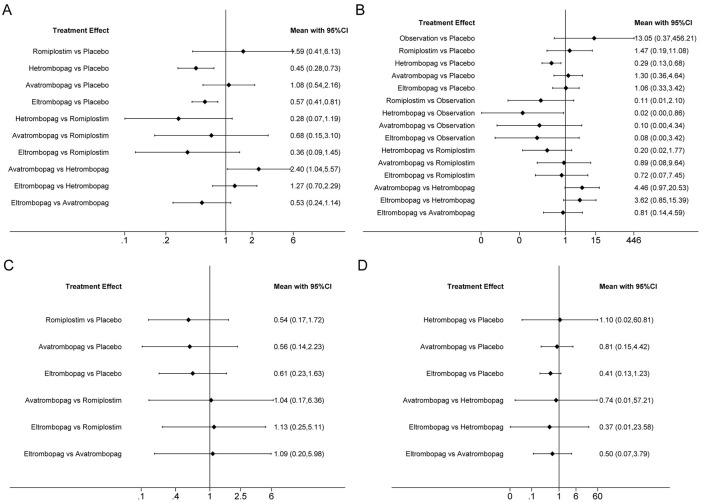
Forest plot of all possible pairwise comparisons for efficacy outcomes. **(A)** Chemotherapy dose reduction or delay due to thrombocytopenia, **(B)** platelet transfusions, **(C)** incidence of grade 3 or 4 thrombocytopenia, and **(D)** bleeding events.

Network plots for each outcome are presented in Figure 3. Five studies reported chemotherapy dose reduction or delay due to thrombocytopenia outcomes. Data from these five studies included four direct comparisons among the five treatments (Figure 3A). As shown in Figure 4A, the results of the network meta-analysis indicate that hetrombopag (summary RR 0.45, 95% confidence interval 0.28–0.73) significantly reduced the incidence of chemotherapy dose reduction or delay due to thrombocytopenia when compared with the placebo, followed by eltrombopag (0.57, 0.41–0.81). However, romiplostim (1.59, 0.41–6.13) increased chemotherapy dose reduction or delay due to thrombocytopenia compared with the placebo. No significant differences were observed between avatrombopag and placebo (1.08, 0.45–2.16). More networks of comparisons can be viewed in Supplementary Table S1. The rankograms are shown in Supplementary Figure S1A. Hetrombopag was ranked as the best treatment for avoiding chemotherapy dose reduction or delay due to thrombocytopenia.

### Platelet transfusions

The network meta-analysis of platelet transfusions included six trials of eltrombopag, avatrombopag, romiplostim, or hetrombopag involving 520 patients (Figure 3B). The results indicate that hetrombopag (0.29, 0.13–0.68) significantly reduced the platelet transfusions compared with the placebo (Figure 4B). However, observation (13.05, 0.37–456.21) increased platelet transfusions compared with the placebo, followed by romiplostim (1.47, 0.19–11.08), avatrombopag (1.30, 0.36–4.64), and eltrombopag (1.06, 0.33–3.42). More networks of comparisons can be viewed in Supplementary Table S2. According to the rankograms (Supplementary Figure S1B), hetrombopag was ranked as the best treatment for reducing platelet transfusions.

### Grade 3 or 4 thrombocytopenia

The network meta-analysis of grade 3 or 4 thrombocytopenia included five trials of eltrombopag, avatrombopag, or romiplostim involving 303 patients (Figure 3C). The pooled results (Figure 4C) demonstrated that these three TPO-RAs greatly reduced the incidence of grade 3 or 4 thrombocytopenia. All possible pairwise comparisons were made, which indicated that romiplostim (0.54, 0.17–1.72) had the lowest risk for grade 3 or 4 thrombocytopenia when compared with the placebo, followed by avatrombopag (0.56, 0.14–2.23) and eltrombopag (0.61, 0.23–1.63). More networks of comparisons can be viewed in Supplementary Table S3. However, none of the comparisons were statistically significant. According to the rankograms (Supplementary Figure S1C), romiplostim was ranked as the best treatment for reducing the incidence of grade 3 or 4 thrombocytopenia.

### Bleeding events

Network plots for bleeding events outcome are presented in Figure 3D. Four studies of eltrombopag, avatrombopag, or hetrombopag with 436 participants reported bleeding events. The pooled results (Figure 4D) demonstrated that eltrombopag (0.41, 0.13–1.23) had the lowest risk for bleeding events when compared with the placebo, followed by avatrombopag (0.81, 0.15–4.42). No significant differences were observed between hetrombopag and placebo (1.10, 0.02–60.81). More networks of comparisons can be viewed in Supplementary Table S4. According to the rankograms (Supplementary Figure S1D), eltrombopag was ranked as the best treatment for avoiding bleeding events.

### Platelet count >400 × 10^9^/L

**FIGURE 5:**
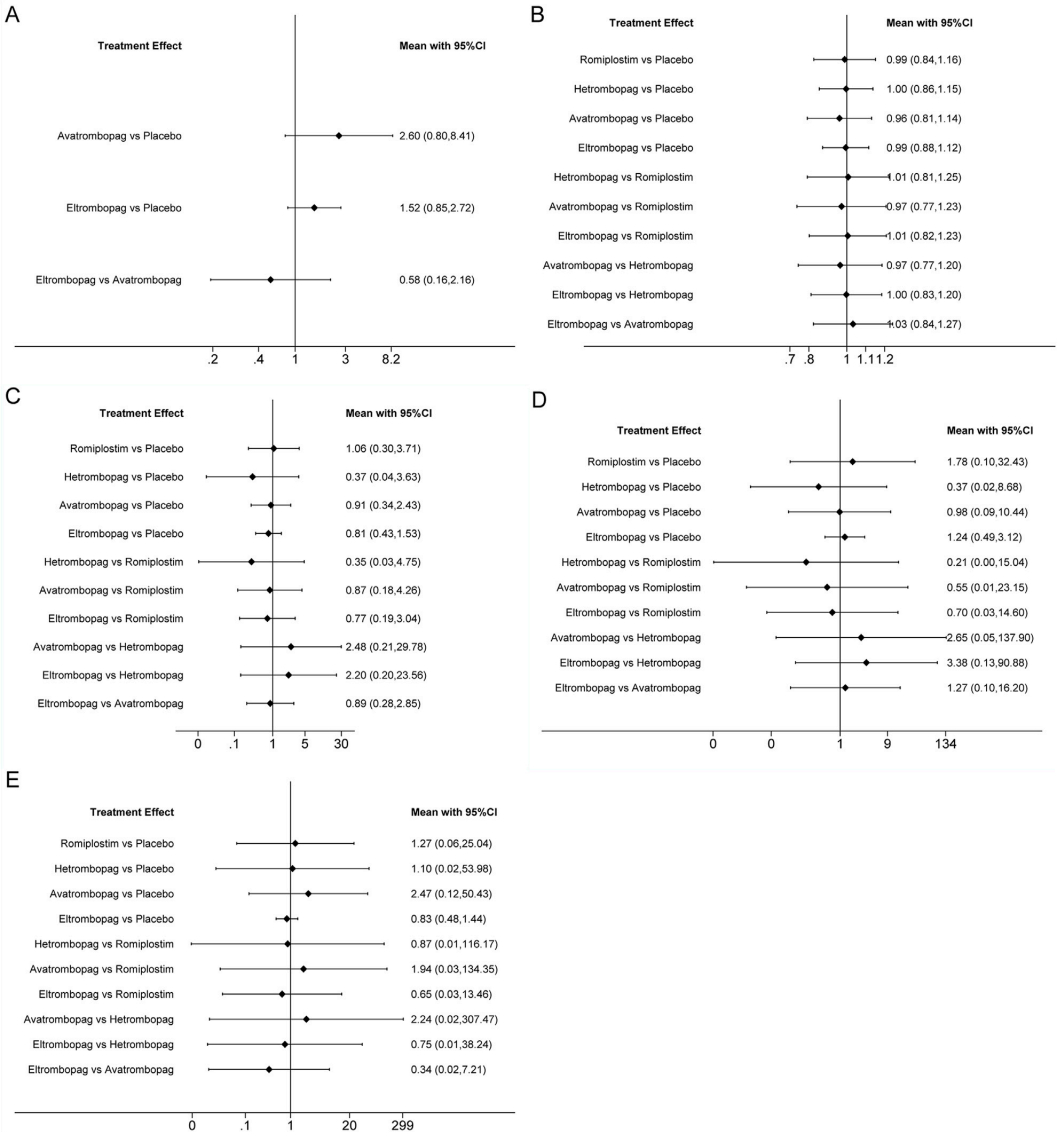
Forest plot of all possible pairwise comparisons for safety outcomes. **(A)** Platelet count > 400 × 10/L, **(B)** AES, **(C)** serious AES, **(D)** thrombosis, and **(E)** mortality.

The network meta-analysis of platelet count >400 × 10^9^/L included three trials of eltrombopag or avatrombopag, involving 328 patients (Figure 3E). The pooled results (Figure 5A) demonstrated that avatrombopag (2.60, 0.80–8.41) and eltrombopag (1.52, 0.85–2.72) increased the incidence of platelet count >400 × 10^9^/L. In addition, avatrombopag showed higher risk for platelet count >400 × 10^9^/L than eltrombopag (1.71, 0.46–6.34). The net league of platelet count >400 × 10^9^/L is shown in Supplementary Table S5. According to the rankograms (Supplementary Figure S2A), placebo was ranked as the best treatment for reducing the incidence of platelet count >400 × 10^9^/L.

### Adverse events

The network meta-analysis of AEs included six trials of eltrombopag, avatrombopag, romiplostim, or hetrombopag involving 524 patients (Figure 3F). When compared with the placebo, there were no significant differences in the risk of AEs between the TPO-RAs, including eltrombopag, avatrombopag, romiplostim, or hetrombopag, with a pooled RR of (0.96, 0.81–1.14), (0.99, 0.84–1.16), (0.99, 0.88–1.12), and (1.00, 0.86–1.15), respectively (Figure 5B). In addition, the pooled data showed no significant differences in AEs between patients receiving the four TPO-RAs. More networks of comparisons can be viewed in Supplementary Table S6. According to the rankograms (Supplementary Figure S2B), avatrombopag carries the least risk of AEs.

### Serious adverse events

The network meta-analysis of serious AEs included seven trials of eltrombopag, avatrombopag, romiplostim, or hetrombopag involving 545 patients (Figure 3G). Compared with the placebo, hetrombopag (0.37, 0.04–3.63) showed the lowest risk of serious AEs, followed by eltrombopag (0.81, 0.43–1.53) and then avatrombopag (0.91, 0.34–2.43) (Figure 5C). No obvious differences in the risk of serious AEs were observed between romiplostim and placebo (1.06, 0.30–3.71). Meanwhile, avatrombopag (2.48, 0.21–29.78) and eltrombopag (2.20, 0.20–23.56) showed higher risk for serious AEs than hetrombopag. However, none of the comparisons were statistically significant. More networks of comparisons can be viewed in Supplementary Table S7. According to the rankograms (Supplementary Figure S2C), hetrombopag carries the least risk of serious AEs.

### Thrombosis

The network meta-analysis of thrombosis included six trials of eltrombopag, avatrombopag, romiplostim, or hetrombopag involving 524 patients (Figure 3H). All possible pairwise comparisons were made (Figure 5D); romiplostim (1.78, 0.10–32.43) and eltrombopag (1.24, 0.49–3.12) may increase the risk of thrombosis compared to the placebo. Meanwhile, hetrombopag (0.37, 0.02–8.68) was found to have the least risk of thrombosis compared to the placebo. The risk of thrombosis associated with avatrombopag is comparable to that with placebo (0.98, 0.09–10.44). Meanwhile, eltrombopag (3.38, 0.13–90.88) and avatrombopag (2.65, 0.05–137.9) showed higher risk of thrombosis than hetrombopag. However, none of the comparisons were statistically significant. More networks of comparisons can be viewed in Supplementary Table S8. According to the rankograms (Supplementary Figure S2D), hetrombopag carries the least risk of thrombosis.

### Mortality

The network meta-analysis of mortality included six trials of eltrombopag, avatrombopag, romiplostim, or hetrombopag involving 524 patients (Figure 3I). All possible pairwise comparisons were made (Figure 5E), and avatrombopag (2.47, 0.12–50.43) was found to have the highest risk of mortality compared to the placebo, followed by romiplostim (1.27, 0.06–25.04) and hetrombopag (1.10, 0.02–53.98). Meanwhile, eltrombopag may reduce mortality when compared to the placebo (0.83, 0.48–1.44), avatrombopag (0.34, 0.02–7.21), romiplostim (0.65, 0.03–13.46), or hetrombopag (0.75, 0.01–38.24). However, none of the comparisons were statistically significant. More networks of comparisons can be viewed in Supplementary Table S9. According to the rankograms (Supplementary Figure S2E), eltrombopag carries the least risk of mortality.

### Additional analyses

The evaluation of the certainty of evidence of outcome measures using the CINeMA indicated that all the evidence was categorized as low or very low (Supplementary Material Appendix 3). Regarding the incoherence domain, effect estimates based only on direct evidence or indirect evidence are assigned a judgment determined by the p-value of the design-by-treatment interaction test. If the design-by-treatment interaction test is not estimable (because the network does not have any closed loop of evidence), then “major concerns” are assigned to all comparisons. The incoherence of all outcomes is “major concerns” because the network does not have any closed loop of evidence. Due to the small number of eligible trials, we could not perform a test for publication bias.

## Discussion

This systematic review and network meta-analysis of TPO-RAs for CIT in solid tumors included dada from eight RCTs including 568 patients who were randomized to romiplostim, eltrombopag, avatrombopag, hetrombopag, placebo, or observation. The quality of the evidence was typically of low risk of bias (seven out of eight trials; 87.5%). Lacking direct comparative data, we performed an indirect comparison to assess the clinical efficacy and safety of these four TPO-RAs in solid tumors with CIT. Low or very low certainty of the evidence evaluated via CINeMA stems largely from incoherence. Without any closed loop of evidence in the network, the incoherence domain was assigned as “major concerns” in all comparisons. Our network meta-analysis compares and ranks the efficacy and safety profiles of different TPO-RAs for CIT among patients with solid tumors to provide more evidence for treatment decision-making.

In general, chemotherapy is administered when pretreatment platelet counts are above 100 × 10^9^/L although many chemotherapy regimens and physicians face challenges when platelet counts fall below 70 × 10^9^/L, particularly below 50 × 10^9^/L, thereby decreasing the dose intensity and clinical outcome. Bleeding events and the necessity for platelet transfusions in CIT are generally rare, with the exception of patients whose platelet counts fall below 25 × 10^9^/L. In these individuals, bleeding rates escalate markedly, and platelet transfusions become the sole therapeutic option. However, platelet counts below 100 × 10^9^/L still pose a significant challenge (Kuter, 2022). The application of rhIL-11 and rhTPO is limited due to adverse effects such as cardiotoxicity and antidrug antibodies that cross-reacted with endogenous TPO (Tepler et al., 1996; Soff et al., 2019). Previous studies have observed the efficacy and safety of TPO-RAs in solid tumors with CIT although some of the results indicated limited efficacy (Chen et al., 2023; Al-Samkari et al., 2022). Whereas it is clear that all TPO-RAs can increase platelet counts in patients with CIT, the critical question remains whether this increase translates into meaningful clinical benefits, such as maintaining relative dose intensity (RDI), reducing bleeding events, and improving treatment response or survival outcomes. The previous meta-analysis demonstrates that TPO-RAs are tolerable and can reduce grade 3 or 4 thrombocytopenia in solid tumors with CIT. However, TPO-RAs do not show advantages in the main efficacy outcomes, including chemotherapy dose reduction or delays, platelet transfusion, and bleeding evens. New evidence has emerged now. For instance, the study on hetrombopag has observed its favorable therapeutic effects (Qin et al., 2024). The previous meta-analysis only analyzed the overall effect of all of TPO-RAs, including romiplostim, eltrombopag, and avatrombopag compared with the placebo or observation group in solid tumors with CIT, but it did not include hetrombopag (Chen et al., 2023). Now, the differences in efficacy among different TPO-RAs and the absence of comparative data in head-to-head trials pose challenges in clinical choices. Our network meta-analysis provides unified hierarchies of evidence of four TPO-RAs in solid tumors with CIT and provides a reference for clinical selection.

TPO is the primary growth factor that stimulates platelet production. TPO-RAs bind to and activate the TPO receptor, leading to an increase in platelet production (Soff et al., 2019). The primary purpose of TPO-RAs for CIT is to maintain the dose schedule and intensity of chemotherapy when such benefit is thought to outweigh potential risks (Griffiths et al., 2022). Patients with persistent CIT, who present with a platelet count <70 to 100 × 10^9^/L on day 1 of a chemotherapy cycle and cannot be safely treated at full dose and on schedule without intervention, may benefit from TPO-RA support (Al-Samkari, 2024). The results of our network meta-analysis indicate that only hetrombopag and eltrombopag significantly reduce the incidence of chemotherapy dose reduction or delay due to thrombocytopenia when compared with the placebo. Based on the indirect evidence in our network meta-analysis, hetrombopag may represent the most favorable approach for avoiding chemotherapy dose reductions or delays. Romiplostim, eltrombopag, and avatrombopag reduced the incidence of grade 3 or 4 thrombocytopenia. However, none of the three reduced platelet transfusions compared with the placebo. Although the results indicate that hetrombopag significantly reduces platelet transfusions compared with the placebo, it might not reduce the risk of bleeding events.

The safety of TPO-RAs is also a major concern. Our network meta-analysis indicates that there were no significant differences in the risk of AEs between these four TPO-RAs compared with the placebo. Indirect comparative data of these four TPO-RAs show that hetrombopag may represent a treatment strategy with a lower risk of serious AEs. Studies have shown that TPO-RAs may increase the risk of venous thromboembolism (VTE). In addition, it has not been confirmed whether the use of TPO-RAs for CIT increases the risk of VTE in patients with cancer (Soff et al., 2019; Griffiths et al., 2022), and caution is required. Previous studies suggested that TPO-RAs may have a potential for increasing thrombotic risk by elevating platelet counts beyond hemostatic needs and promoting the production of younger, hyperactive platelets (Rodeghiero, 2016). Our network meta-analysis indicates that only eltrombopag and avatrombopag studies reported the data of platelet count >400 × 10^9^/L, and both of them increased in the incidence of platelet count >400 × 10^9^/L compared with placebo. However, they might not have a significantly increased risk of thrombosis. According to indirect evidence in our network meta-analysis, hetrombopag may be the preferred strategy with lower thrombosis risk. Avatrombopag was found to increase the risk of mortality compared to the placebo. Conversely, indirect comparative evidence indicates that eltrombopag was associated with a lower risk of mortality.

Some rare adverse reactions also require attention. Initial concerns were raised about the potential for bone marrow fibrosis due to prolonged stimulation of megakaryopoiesis by TPO-RAs (Kuter et al., 2009). TPO-RAs including romiplostim and eltrombopag have been reported to increase the risk of reticulin fiber deposition within bone marrow (Kuter et al., 2009; Ghanima et al., 2014). However, subsequently, numerous prospective and retrospective studies have shown that in most patients, the grade of fibrosis did not change during treatment with TPO-RA (Brynes et al., 2015; Brynes et al., 2017). Initial concerns regarding myelofibrosis have not been confirmed. Only a minority of patients experience moderate–severe reticulin and/or collagen fibrosis, which usually reverses upon the discontinuation of TPO-RAs (Ghanima et al., 2019). Cataract formation has been observed in patients treated with both eltrombopag and romiplostim. However, due to numerous confounding factors such as corticosteroid use, advanced age, and smoking, no clinical trial has definitively confirmed this potential association with TPO-RAs (Ghanima et al., 2019; Cheng et al., 2011).

A key consideration in interpreting the findings of this network meta-analysis is the clinical heterogeneity between the included trials. For instance, the inclusion criteria were different. In the two studies (Winer et al., 2015; Winer et al., 2017) that reported the efficacy of eltrombopag for CIT, eligible patients were those with platelet counts >100 × 10^9^/L prior to starting eltrombopag or placebo. In addition, in the study about romiplostim, the eligible patients had experienced a transient platelet count decrease to <100 × 10^9^/L in a previous treatment cycle, meaning that patients had recovered from CIT before romiplostim or placebo. Meanwhile, in the study about hetrombopag (Qin et al., 2024) or avatrombopag (Al-Samkari et al., 2022), eligible patients were those with platelet counts <75 × 10^9^/L prior to starting intervention or placebo. Variations in baseline platelet count thresholds, the specific chemotherapeutic regimens used, and the solid tumor types enrolled could substantially influence the observed efficacy and safety outcomes of TPO-RAs. Due to these differences in trial designs, and including the major differences in dosing regimens and outcomes evaluated, the result of this network analysis comparing the differences in efficacy and safety for CIT between the various TPO-RAs needs to be understood dialectically (Al-Samkari et al., 2022).

Although our analysis and existing evidence demonstrate the efficacy of TPO-RAs in increasing the platelet counts, there are several significant practical barriers to the clinical use of TPO-RAs, including the cost, limited availability of the drug (e.g., hetrombopag is currently approved only in China), and the need to monitor for thrombosis during treatment. The clinical value of TPO-RAs may vary depending on the severity and duration of CIT. The typical lifespan of a platelet is 8–10 days. Following many types of chemotherapy, platelet counts usually begin to decline by day 7, reach their lowest point at approximately day 14, and then gradually recover to baseline levels between day 28 and day 35 (Shimazaki et al., 1997). For patients with transient CIT, platelet counts may recover spontaneously to a normal or near-normal platelet count by the start of their next cycle. The pros and cons need to be weighed in the use of TPO-RAs on patients with transient CIT because they usually do not require treatment for CIT unless they develop bleeding in association with the CIT or they have profound nadir thrombocytopenia (typically a platelet count <20–30 × 10^9^/L) (Al-Samkari, 2024). TPO-RAs support may benefit patients with persistent CIT who have a platelet count <70–100 × 10^9^/L on day 1 of the chemotherapy cycle and cannot safely receive the full-dose, on-schedule treatment without intervention (Al-Samkari, 2024; Song and Al-Samkari, 2025).

Future research should prioritize well-designed, prospective RCTs that directly compare these TPO-RAs in patients with severe and/or persistent CIT. Furthermore, conducting an individual patient data network meta-analysis to adjust for covariates such as age, tumor type, and prior chemotherapy cycles would be a valuable endeavor.

## Limitation

Due to the lack of head-to-head studies and the scarce data from a small number of included RCTs, the low or very low evidence based on indirect comparative data needs to be interpreted with caution. Publication bias was the main reason for the small sample effect. As fewer than 10 RCTs (eight) were included in our study, we could not draw a funnel plot to identify publication bias. The high clinical heterogeneity due to differences in the design of the including trials have also limited the interpretation of the results. It is important to consider that these agents are used most of the time to support chemotherapy continuation and or to increase platelet counts above 100 × 10^9^/L. Therefore, other agents that are not known to cause platelet counts >400 × 10^9^/L are likely not associated with such high values because they were discontinued before reaching those levels. Most of trials (6/8) focused only on patients with transient thrombocytopenia and did not evaluate those with more severe or persistent CIT. Due to the limited number of included RCTs and the variability in dosing regimens (e.g., romiplostim doses ranging from 250 to 750 μg), we were unable to perform a meaningful dose–response analysis. Further large-scale, head-to-head RCTs are necessary to evaluate the efficacy and safety of TPO-RAs to manage severe, persistent CIT.

## Conclusion

Our network meta-analysis provides low- to very low-certainty evidence, suggesting that based on the limited indirect data, hetrombopag may represent the preferred therapeutic strategy for avoiding chemotherapy dose reduction or delay and reducing platelet transfusion requirements, whereas eltrombopag stood out for reducing the risk of bleeding events and mortality, with both demonstrating acceptable safety. Further large-scale, prospective, head-to-head trials are needed to determine the safety, efficacy, and benefits of TPO-RAs in managing severe, persistent CIT.

## Data Availability

The original contributions presented in the study are included in the article/Supplementary Material, further inquiries can be directed to the corresponding author.
