# Supplementary material for: The efficacy and safety of thrombopoietin receptor agonists in solid tumors with chemotherapy-induced thrombocytopenia: a systematic review and network meta-analysis of randomized controlled trials
Source: Front Pharmacol. 2025 Dec 1;16:1683857. doi: 10.3389/fphar.2025.1683857 (PMC12702962; doi:10.3389/fphar.2025.1683857)

Supplementary Material

# Appendix 1 Search strategy

**Pubmed**

| ID | Search | Results |
| --- | --- | --- |
| 13 | (("Neoplasms"[MeSH Terms] OR ("benign neoplasm*"[Title/Abstract] OR "cancer*"[Title/Abstract] OR "malignancies"[Title/Abstract] OR "malignancy"[Title/Abstract] OR "malignant neoplas*"[Title/Abstract] OR "malignant tumor"[Title/Abstract] OR "malignant tumour"[Title/Abstract] OR "Malignant Neoplasms"[Title/Abstract] OR "neoplasia*"[Title/Abstract] OR "neoplasm*"[Title/Abstract] OR "neoplastic malignancy"[Title/Abstract] OR "oncological malignancy"[Title/Abstract] OR "tumor*"[Title/Abstract] OR "tumoral malignancy"[Title/Abstract] OR "tumour"[Title/Abstract])) NOT ("Hematologic Neoplasms"[MeSH Terms] OR ("blood cancer*"[Title/Abstract] OR "haematologic malignancy"[Title/Abstract] OR "haematological malignancy"[Title/Abstract] OR "haematological malignant disease"[Title/Abstract] OR "hematologic malignanc*"[Title/Abstract] OR "hematologic malignant disease"[Title/Abstract] OR "hematologic neoplasm*"[Title/Abstract] OR "hematological malignanc*"[Title/Abstract] OR "hematological malignant disease"[Title/Abstract] OR "hematological malignant disorder"[Title/Abstract] OR "hematological neoplasm*"[Title/Abstract] OR "hematopoietic malignanc*"[Title/Abstract] OR "hematopoietic neoplasm*"[Title/Abstract] OR "malignant blood disease"[Title/Abstract] OR "malignant blood disorder"[Title/Abstract] OR "malignant haematologic disease"[Title/Abstract] OR "malignant haematological disease"[Title/Abstract] OR "malignant haematological disorder"[Title/Abstract] OR "malignant hematologic disease"[Title/Abstract] OR "malignant hematologic disorder"[Title/Abstract] OR "malignant hematological disease"[Title/Abstract] OR "malignant hematological disorder"[Title/Abstract]))) AND ("Thrombocytopenia"[MeSH Terms] OR ("macrothrombocytopaenia"[Title/Abstract] OR "macrothrombocytopenia"[Title/Abstract] OR "platelet count decreased"[Title/Abstract] OR "platelet deficiency"[Title/Abstract] OR "thrombocytopaenia"[Title/Abstract] OR "thrombocytopenia*"[Title/Abstract] OR "thrombopenia*"[Title/Abstract])) AND ("thrombopoietin receptor agonist*"[Title/Abstract] OR "tpo ra*"[Title/Abstract] OR "Hetrombopag"[Title/Abstract] OR "Avatrombopag"[Title/Abstract] OR "Lusutrombopag"[Title/Abstract] OR "Eltrombopag"[Title/Abstract] OR "Romiplostim"[Title/Abstract]) AND (("randomized controlled trial"[Publication Type] OR "controlled clinical trial"[Publication Type] OR "randomized"[Title/Abstract] OR "placebo"[Title/Abstract] OR "drug therapy"[MeSH Subheading] OR "randomly"[Title/Abstract] OR "trial"[Title/Abstract] OR "groups"[Title/Abstract]) NOT ("animals"[MeSH Terms] NOT "humans"[MeSH Terms])) | **165** |
| 12 | ("Neoplasms"[MeSH Terms] OR ("benign neoplasm*"[Title/Abstract] OR "cancer*"[Title/Abstract] OR "malignancies"[Title/Abstract] OR "malignancy"[Title/Abstract] OR "malignant neoplas*"[Title/Abstract] OR "malignant tumor"[Title/Abstract] OR "malignant tumour"[Title/Abstract] OR "Malignant Neoplasms"[Title/Abstract] OR "neoplasia*"[Title/Abstract] OR "neoplasm*"[Title/Abstract] OR "neoplastic malignancy"[Title/Abstract] OR "oncological malignancy"[Title/Abstract] OR "tumor*"[Title/Abstract] OR "tumoral malignancy"[Title/Abstract] OR "tumour"[Title/Abstract])) NOT ("Hematologic Neoplasms"[MeSH Terms] OR ("blood cancer*"[Title/Abstract] OR "haematologic malignancy"[Title/Abstract] OR "haematological malignancy"[Title/Abstract] OR "haematological malignant disease"[Title/Abstract] OR "hematologic malignanc*"[Title/Abstract] OR "hematologic malignant disease"[Title/Abstract] OR "hematologic neoplasm*"[Title/Abstract] OR "hematological malignanc*"[Title/Abstract] OR "hematological malignant disease"[Title/Abstract] OR "hematological malignant disorder"[Title/Abstract] OR "hematological neoplasm*"[Title/Abstract] OR "hematopoietic malignanc*"[Title/Abstract] OR "hematopoietic neoplasm*"[Title/Abstract] OR "malignant blood disease"[Title/Abstract] OR "malignant blood disorder"[Title/Abstract] OR "malignant haematologic disease"[Title/Abstract] OR "malignant haematological disease"[Title/Abstract] OR "malignant haematological disorder"[Title/Abstract] OR "malignant hematologic disease"[Title/Abstract] OR "malignant hematologic disorder"[Title/Abstract] OR "malignant hematological disease"[Title/Abstract] OR "malignant hematological disorder"[Title/Abstract])) | 5,242,874 |
| 11 | "Thrombocytopenia"[MeSH Terms] OR "macrothrombocytopaenia"[Title/Abstract] OR "macrothrombocytopenia"[Title/Abstract] OR "platelet count decreased"[Title/Abstract] OR "platelet deficiency"[Title/Abstract] OR "thrombocytopaenia"[Title/Abstract] OR "thrombocytopenia*"[Title/Abstract] OR "thrombopenia*"[Title/Abstract] | 92,839 |
| 10 | "Hematologic Neoplasms"[MeSH Terms] OR "blood cancer*"[Title/Abstract] OR "haematologic malignancy"[Title/Abstract] OR "haematological malignancy"[Title/Abstract] OR "haematological malignant disease"[Title/Abstract] OR "hematologic malignanc*"[Title/Abstract] OR "hematologic malignant disease"[Title/Abstract] OR "hematologic neoplasm*"[Title/Abstract] OR "hematological malignanc*"[Title/Abstract] OR "hematological malignant disease"[Title/Abstract] OR "hematological malignant disorder"[Title/Abstract] OR "hematological neoplasm*"[Title/Abstract] OR "hematopoietic malignanc*"[Title/Abstract] OR "hematopoietic neoplasm*"[Title/Abstract] OR "malignant blood disease"[Title/Abstract] OR "malignant blood disorder"[Title/Abstract] OR "malignant haematologic disease"[Title/Abstract] OR "malignant haematological disease"[Title/Abstract] OR "malignant haematological disorder"[Title/Abstract] OR "malignant hematologic disease"[Title/Abstract] OR "malignant hematologic disorder"[Title/Abstract] OR "malignant hematological disease"[Title/Abstract] OR "malignant hematological disorder"[Title/Abstract] | 56,217 |
| 9 | "Neoplasms"[MeSH Terms] OR "benign neoplasm*"[Title/Abstract] OR "cancer*"[Title/Abstract] OR "malignancies"[Title/Abstract] OR "malignancy"[Title/Abstract] OR "malignant neoplas*"[Title/Abstract] OR "malignant tumor"[Title/Abstract] OR "malignant tumour"[Title/Abstract] OR "Malignant Neoplasms"[Title/Abstract] OR "neoplasia*"[Title/Abstract] OR "neoplasm*"[Title/Abstract] OR "neoplastic malignancy"[Title/Abstract] OR "oncological malignancy"[Title/Abstract] OR "tumor*"[Title/Abstract] OR "tumoral malignancy"[Title/Abstract] OR "tumour"[Title/Abstract] | 5,298,965 |
| 8 | ("randomized controlled trial"[Publication Type] OR "controlled clinical trial"[Publication Type] OR "randomized"[Title/Abstract] OR "placebo"[Title/Abstract] OR "drug therapy"[MeSH Subheading] OR "randomly"[Title/Abstract] OR "trial"[Title/Abstract] OR "groups"[Title/Abstract]) NOT ("animals"[MeSH Terms] NOT "humans"[MeSH Terms]) | 5,478,539 |
| 7 | "thrombopoietin receptor agonist*"[Title/Abstract] OR "tpo ra*"[Title/Abstract] OR "Hetrombopag"[Title/Abstract] OR "Avatrombopag"[Title/Abstract] OR "Lusutrombopag"[Title/Abstract] OR "Eltrombopag"[Title/Abstract] OR "Romiplostim"[Title/Abstract] | 2,279 |
| 6 | "macrothrombocytopaenia"[Title/Abstract] OR "macrothrombocytopenia"[Title/Abstract] OR "platelet count decreased"[Title/Abstract] OR "platelet deficiency"[Title/Abstract] OR "thrombocytopaenia"[Title/Abstract] OR "thrombocytopenia*"[Title/Abstract] OR "thrombopenia*"[Title/Abstract] | 66,462 |
| 5 | "Thrombocytopenia"[MeSH Terms] | 55,473 |
| 4 | "blood cancer*"[Title/Abstract] OR "haematologic malignancy"[Title/Abstract] OR "haematological malignancy"[Title/Abstract] OR "haematological malignant disease"[Title/Abstract] OR "hematologic malignanc*"[Title/Abstract] OR "hematologic malignant disease"[Title/Abstract] OR "hematologic neoplasm*"[Title/Abstract] OR "hematological malignanc*"[Title/Abstract] OR "hematological malignant disease"[Title/Abstract] OR "hematological malignant disorder"[Title/Abstract] OR "hematological neoplasm*"[Title/Abstract] OR "hematopoietic malignanc*"[Title/Abstract] OR "hematopoietic neoplasm*"[Title/Abstract] OR "malignant blood disease"[Title/Abstract] OR "malignant blood disorder"[Title/Abstract] OR "malignant haematologic disease"[Title/Abstract] OR "malignant haematological disease"[Title/Abstract] OR "malignant haematological disorder"[Title/Abstract] OR "malignant hematologic disease"[Title/Abstract] OR "malignant hematologic disorder"[Title/Abstract] OR "malignant hematological disease"[Title/Abstract] OR "malignant hematological disorder"[Title/Abstract] | 39,632 |
| 3 | "Hematologic Neoplasms"[MeSH Terms] | 26,750 |
| 2 | "benign neoplasm*"[Title/Abstract] OR "cancer*"[Title/Abstract] OR "malignancies"[Title/Abstract] OR "malignancy"[Title/Abstract] OR "malignant neoplas*"[Title/Abstract] OR "malignant tumor"[Title/Abstract] OR "malignant tumour"[Title/Abstract] OR "Malignant Neoplasms"[Title/Abstract] OR "neoplasia*"[Title/Abstract] OR "neoplasm*"[Title/Abstract] OR "neoplastic malignancy"[Title/Abstract] OR "oncological malignancy"[Title/Abstract] OR "tumor*"[Title/Abstract] OR "tumoral malignancy"[Title/Abstract] OR "tumour"[Title/Abstract] | 3,954,682 |
| 1 | "Neoplasms"[MeSH Terms] | 4,055,180 |

**Cochrane Library**

| ID | Search | Results |
| --- | --- | --- |
| #1 | MeSH descriptor: [Neoplasms] explode all trees | 128820 |
| #2 | ((‘benign neoplasm*’ OR ‘cancer*’ OR ‘malignancies’ OR ‘malignancy’ OR ‘malignant neoplas*’ OR ‘malignant tumor’ OR ‘malignant tumour’ OR ‘Malignant Neoplasms’ OR ‘neoplasia*’ OR ‘neoplasm*’ OR ‘neoplasmic malignancy’ OR ‘neoplastic malignancy’ OR ‘oncologic malignancy’ OR ‘oncological malignancy’ OR ‘tumor*’ OR ‘tumoral malignancy’ OR ‘tumorous malignancy’ OR ‘tumour’)):ti,ab,kw | 279158 |
| #3 | #1 OR #2 | 292898 |
| #4 | MeSH descriptor: [Hematologic Neoplasms] explode all trees | 983 |
| #5 | (‘blood cancer*’ OR ‘haematologic malignancy’ OR ‘haematologic malignant disease’ OR ‘haematologic malignant disorder’ OR ‘haematological malignancy’ OR ‘haematological malignant disease’ OR ‘haematological malignant disorder’ OR ‘hematologic malignanc*’ OR ‘hematologic malignant disease’ OR ‘hematologic malignant disorder’ OR ‘hematologic neoplasm*’ OR ‘hematological malignanc*’ OR ‘hematological malignant disease’ OR ‘hematological malignant disorder’ OR ‘hematological neoplasm*’ OR ‘hematopoietic malignanc*’ OR ‘hematopoietic neoplasm*’ OR ‘malignant blood disease’ OR ‘malignant blood disorder’ OR ‘malignant haematologic disease’ OR ‘malignant haematologic disorder’ OR ‘malignant haematological disease’ OR ‘malignant haematological disorder’ OR ‘malignant haematological illness’ OR ‘malignant hematologic disease’ OR ‘malignant hematologic disorder’ OR ‘malignant hematological disease’ OR ‘malignant hematological disorder’):ti,ab,kw | 43382 |
| #6 | #4 OR #5 | 43529 |
| #7 | MeSH descriptor: [Thrombocytopenia] explode all trees | 1833 |
| #8 | (‘macrothrombocytopaenia’ OR ‘macrothrombocytopenia’ OR ‘platelet count decreased’ OR ‘platelet deficiency’ OR ‘thrombocyte deficiency’ OR ‘thrombocytopaenia’ OR ‘thrombocytopenia*’ OR ‘thrombopenia*’):ti,ab,kw | 13495 |
| #9 | #7 OR #8 | 13736 |
| #10 | (‘thrombopoietin receptor agonist*’ OR ‘TPO-Ra*’ OR ‘Hetrombopag ‘ OR ‘Avatrombopag’ OR ‘Lusutrombopag’ OR ‘Eltrombopag’ OR ‘Romiplostim’):ti,ab,kw | 745 |
| #11 | #3 NOT #6 | 249733 |
| #12 | #11 AND #9 AND #10 | **83(trials 82)** |

**Embase**

| ID | Search | Results |
| --- | --- | --- |
| #13 | #7 AND #10 AND #11 AND #12 | **74** |
| #12 | 'randomized controlled trial'/exp OR 'randomized':ti,ab,kw OR 'randomised controlled study':ti,ab,kw OR 'randomised controlled trial':ti,ab,kw OR 'placebo':ti,ab,kw OR 'randomly':ti,ab,kw | 1899052 |
| #11 | 'thrombopoietin receptor agonist*':ti,ab,kw OR 'tpo-ra*':ti,ab,kw OR 'hetrombopag':ti,ab,kw OR 'avatrombopag':ti,ab,kw OR 'lusutrombopag':ti,ab,kw OR 'eltrombopag':ti,ab,kw OR 'romiplostim':ti,ab,kw | 4873 |
| #10 | #8 OR #9 | 265212 |
| #9 | 'macrothrombocytopaenia':ti,ab,kw OR 'macrothrombocytopenia':ti,ab,kw OR 'platelet count decreased':ti,ab,kw OR 'platelet deficiency':ti,ab,kw OR 'thrombocyte deficiency':ti,ab,kw OR 'thrombocytopaenia':ti,ab,kw OR 'thrombocytopenia*':ti,ab,kw OR 'thrombopenia*':ti,ab,kw | 118828 |
| #8 | 'thrombocytopenia'/exp | 249862 |
| #7 | #3 NOT #6 | 5867251 |
| #6 | #4 OR #5 | 930786 |
| #5 | 'blood cancer*':ti,ab,kw OR 'haematologic malignancy':ti,ab,kw OR 'haematologic malignant disease':ti,ab,kw OR 'haematologic malignant disorder':ti,ab,kw OR 'haematological malignancy':ti,ab,kw OR 'haematological malignant disease':ti,ab,kw OR 'haematological malignant disorder':ti,ab,kw OR 'hematologic malignanc*':ti,ab,kw OR 'hematologic malignant disease':ti,ab,kw OR 'hematologic malignant disorder':ti,ab,kw OR 'hematologic neoplasm*':ti,ab,kw OR 'hematological malignanc*':ti,ab,kw OR 'hematological malignant disease':ti,ab,kw OR 'hematological malignant disorder':ti,ab,kw OR 'hematological neoplasm*':ti,ab,kw OR 'hematopoietic malignanc*':ti,ab,kw OR 'hematopoietic neoplasm*':ti,ab,kw OR 'malignant blood disease':ti,ab,kw OR 'malignant blood disorder':ti,ab,kw OR 'malignant haematologic disease':ti,ab,kw OR 'malignant haematologic disorder':ti,ab,kw OR 'malignant haematological disease':ti,ab,kw OR 'malignant haematological disorder':ti,ab,kw OR 'malignant haematological illness':ti,ab,kw OR 'malignant hematologic disease':ti,ab,kw OR 'malignant hematologic disorder':ti,ab,kw OR 'malignant hematological disease':ti,ab,kw OR 'malignant hematological disorder':ti,ab,kw | 72773 |
| #4 | 'hematologic malignancy'/exp | 915769 |
| #3 | #1 OR #2 | 6797879 |
| #2 | 'benign neoplasm*':ti,ab,kw OR 'cancer*':ti,ab,kw OR 'malignancies':ti,ab,kw OR 'malignancy':ti,ab,kw OR 'malignant neoplas*':ti,ab,kw OR 'malignant tumor':ti,ab,kw OR 'malignant tumour':ti,ab,kw OR 'malignant neoplasms':ti,ab,kw OR 'neoplasia*':ti,ab,kw OR 'neoplasm*':ti,ab,kw OR 'neoplasmic malignancy':ti,ab,kw OR 'neoplastic malignancy':ti,ab,kw OR 'oncologic malignancy':ti,ab,kw OR 'oncological malignancy':ti,ab,kw OR 'tumor*':ti,ab,kw OR 'tumoral malignancy':ti,ab,kw OR 'tumorous malignancy':ti,ab,kw OR 'tumour':ti,ab,kw | 5461636 |
| #1 | 'malignant neoplasm'/exp | 4892883 |

**Ovid MEDLINE**

| ID | Search | Results |
| --- | --- | --- |
| 1 | Neoplasms/ | 534209 |
| 2 | (randomized controlled trial or controlled clinical trial).pt. | 720070 |
| 3 | clinical trials as topic/ | 204105 |
| 4 | (random* or placebo*).ti,ab. | 1673399 |
| 5 | trial.ti. | 325821 |
| 6 | 2 or 3 or 4 or 5 | 2068068 |
| 7 | (benign neoplasm* or cancer* or malignancies or malignancy or malignant neoplas* or malignant tumor or malignant tumour neoplasia* or neoplasm* or neoplasmic malignancy or neoplastic malignancy or oncologic malignancy or oncological malignancy or tumor* or tumoral malignancy or tumorous malignancy or tumour).ti,ab,kw. | 3854474 |
| 8 | (blood cancer* or haematologic malignancy or haematologic malignant disease or haematologic malignant disorder or haematological malignancy or haematological malignant disease or haematological malignant disorder or hematologic malignanc* or hematologic malignant disease or hematologic malignant disorder or hematologic neoplasm* or hematological malignanc* or hematological malignant disease or hematological malignant disorder or hematological neoplasm* or hematopoietic malignanc* or hematopoietic neoplasm* or malignant blood disease or malignant blood disorder or malignant haematologic disease or malignant haematologic disorder or malignant haematological disease or malignant haematological disorder or malignant haematological illness or malignant hematologic disease or malignant hematologic disorder or malignant hematological disease or malignant hematological disorder).ab,kw,ti. | 39664 |
| 9 | Hematologic Neoplasms/ | 18163 |
| 10 | Thrombocytopenia/ | 31379 |
| 11 | (macrothrombocytopaenia or macrothrombocytopenia or platelet count decreased or platelet deficiency or thrombocyte deficiency or thrombocytopaenia or thrombocytopenia* or thrombopenia*).ab,kw,ti. | 66259 |
| 12 | (thrombopoietin receptor agonist* or TPO-Ra* or Hetrombopag or Avatrombopag or Lusutrombopag or Eltrombopag or Romiplostim).ab,kw,ti. | 2300 |
| 13 | 1 or 7 | 3951776 |
| 14 | 8 or 9 | 47781 |
| 15 | 13 not 14 | 3908239 |
| 16 | 10 or 11 | 75513 |
| 17 | 6 and 12 and 15 and 16 | **31** |

**Web of Science**

| ID | Search | Results |
| --- | --- | --- |
| 1 | TS=(“benign neoplasm*” OR “cancer*” OR “malignancies” OR “malignancy” OR “malignant neoplas*” OR “malignant tumor” OR “malignant tumour” OR “Malignant Neoplasms” OR “neoplasia*” OR “neoplasm*” OR “neoplasmic malignancy” OR “neoplastic malignancy” OR “oncologic malignancy” OR “oncological malignancy” OR “tumor*” OR “tumoral malignancy” OR “tumorous malignancy” OR “tumour”) | 4849523 |
| 2 | TS=(“blood cancer*” OR “haematologic malignancy” OR “haematologic malignant disease” OR “haematologic malignant disorder” OR “haematological malignancy” OR “haematological malignant disease” OR “haematological malignant disorder” OR “hematologic malignanc*” OR “hematologic malignant disease” OR “hematologic malignant disorder” OR “hematologic neoplasm*” OR “hematological malignanc*” OR “hematological malignant disease” OR “hematological malignant disorder” OR “hematological neoplasm*” OR “hematopoietic malignanc*” OR “hematopoietic neoplasm*” OR “malignant blood disease” OR “malignant blood disorder” OR “malignant haematologic disease” OR “malignant haematologic disorder” OR “malignant haematological disease” OR “malignant haematological disorder” OR “malignant haematological illness” OR “malignant hematologic disease” OR “malignant hematologic disorder” OR “malignant hematological disease” OR “malignant hematological disorder”) | 48340 |
| 3 | TS=(“macrothrombocytopaenia” OR “macrothrombocytopenia” OR “platelet count decreased” OR “platelet deficiency” OR “thrombocyte deficiency” OR “thrombocytopaenia” OR “thrombocytopenia*” OR “thrombopenia*”) | 69689 |
| 4 | TS=(“thrombopoietin receptor agonist*” OR “TPO-Ra*” OR “Hetrombopag “ OR “Avatrombopag” OR “Lusutrombopag” OR “Eltrombopag” OR “Romiplostim”) | 3499 |
| 5 | TI=(random* OR placebo* OR trial) | 902607 |
| 6 | AB=(random* OR placebo*) | 2306054 |
| 7 | #6 OR #5 | 2748155 |
| 8 | #1 NOT #2 | 4801435 |
| 9 | #8 AND #3 AND #4 AND #7 | **42** |

**ClinialTrials.gov**

| ID | Search | Results |
| --- | --- | --- |
| 1 | Neoplasms \| “thrombopoietin receptor agonist*” OR “TPO-Ra*” OR “Hetrombopag “ OR “Avatrombopag” OR “Lusutrombopag” OR “Eltrombopag” OR “Romiplostim” | **86** |

**CNKI**

| ID | Search | Results |
| --- | --- | --- |
| 1 | （篇关摘：肿瘤+癌+瘤（精确））NOT（篇关摘：血液肿瘤（精确））AND（篇关摘：血小板减少（精确））AND（篇关摘：血小板生成素受体激动剂+'TPO-RA'’+海曲泊帕+阿伐曲泊帕+芦曲波帕+艾曲波帕+罗普司亭（精确））AND（篇关摘：随机对照试验+随机对照实验+随机对照研究（精确）） | **3** |

**Wanfang Database**

| ID | Search | Results |
| --- | --- | --- |
| 1 | （中英文扩展&主题词扩展）：主题:(肿瘤 or 癌 or 瘤) not 主题:(血液肿瘤) and 主题:(血小板减少) and 主题:(血小板生成素受体激动剂 or \‘TPO\-RA\’ or 海曲泊帕 or 阿伐曲泊帕 or 芦曲波帕 or 艾曲波帕 or 罗普司亭) and 主题:(随机对照试验 or 随机对照实验 or 随机对照研究) | **7** |

**VIP**

| ID | Search | Results |
| --- | --- | --- |
| 1 | （（（（（（任意字段=肿瘤OR 任意字段=癌）OR 任意字段=瘤）AND（NOT 任意字段=血液肿瘤））AND 任意字段=血小板减少）AND（（（（（（任意字段=血小板生成素受体激动剂 OR（任意字段=TPO AND（NOT 任意字段=RA）））OR 任意字段=海曲泊帕）OR 任意字段=阿伐曲泊帕）OR 任意字段=芦曲波帕）OR 任意字段=艾曲波帕）OR 任意字段=罗普司亭））AND（（任意字段=随机对照试验OR任意字段=随机对照实验）OR 任意字段=随机对照研究）） | **2** |

**SinoMed**

| ID | Search | Results |
| --- | --- | --- |
| 1 | ( "肿瘤"[常用字段:智能] OR "癌"[常用字段:智能] OR "瘤"[常用字段:智能]) | 2185579 |
| 2 | "血液肿瘤"[常用字段:智能] | 9450 |
| 3 | "血小板减少"[常用字段:智能] | 31709 |
| 4 | ( "血小板生成素受体激动剂"[常用字段:智能] OR "‘TPO-RA’"[常用字段:智能] OR "海曲泊帕"[常用字段:智能] OR "阿伐曲泊帕"[常用字段:智能] OR "芦曲波帕"[常用字段:智能] OR "艾曲波帕"[常用字段:智能] OR "罗普司亭"[常用字段:智能]) | 277 |
| 5 | ( "随机对照试验"[常用字段:智能] OR "随机对照实验"[常用字段:智能] OR "随机对照研究"[常用字段:智能]) | 226504 |
| 6 | (#2) NOT (#3) | 2176851 |
| 7 | (#8) AND (#6) AND (#5) AND (#4) | **1** |

**China drug trials** (www.chinadrugtrials.org.cn)

| ID | Search | Results |
| --- | --- | --- |
| 1 | （（适应症=肿瘤）AND （药物=海曲泊帕OR艾曲波帕OR 阿伐曲泊帕 OR 罗米司亭 OR 芦曲波帕）） | **5** |

# Appendix 2 Supplementary Figures and Tables

## 2.1 Supplementary Figures

##
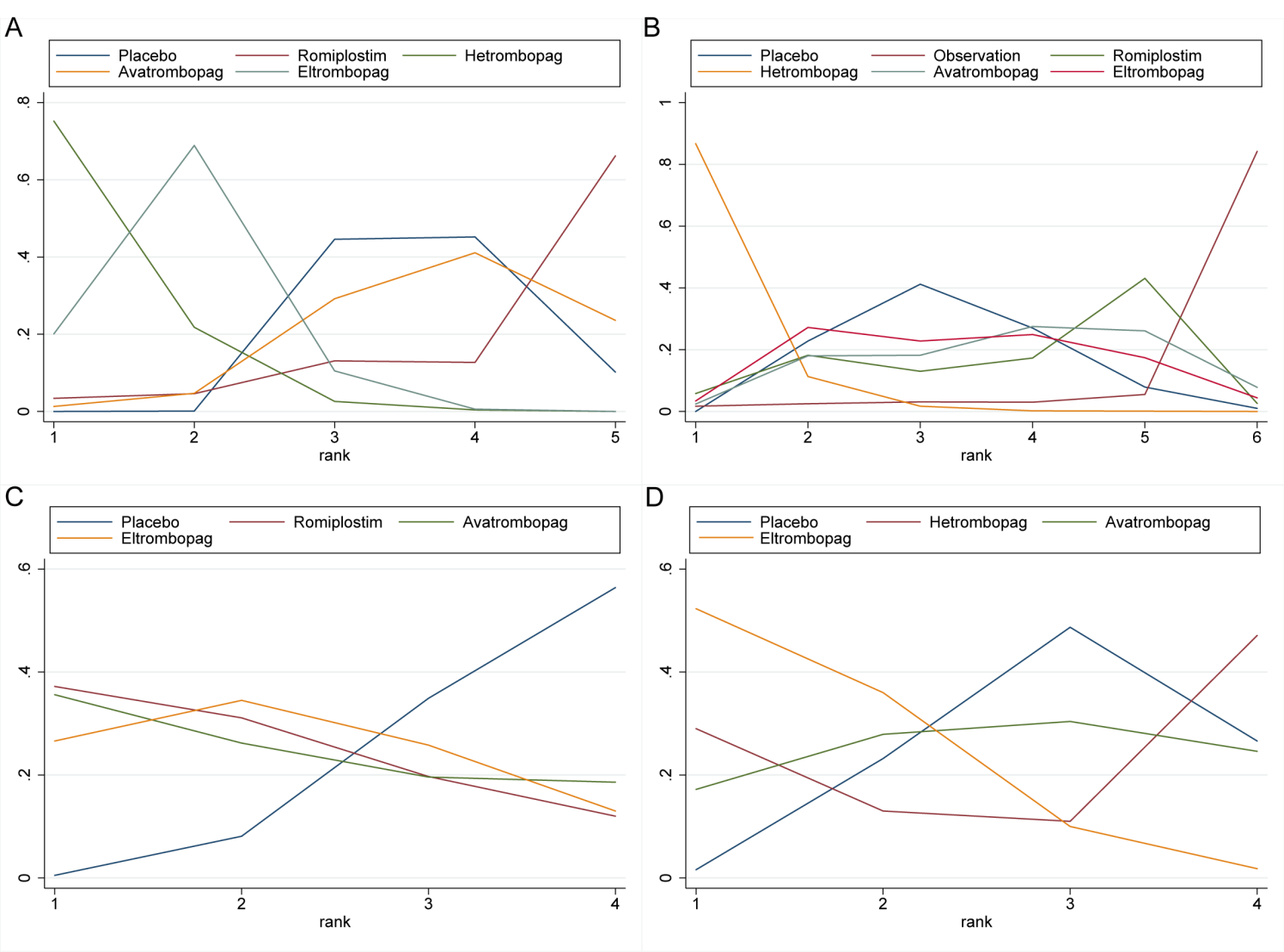


## **Supplementary Figure 1.** Rank probability curves (Rankograms) for efficacy outcomes. **(A)**chemotherapy dose reduction or delay due to thrombocytopenia, **(B)**platelet transfusions, **(C)**incidence of grade 3 or 4 thrombocytopenia, **(D)**bleeding events.


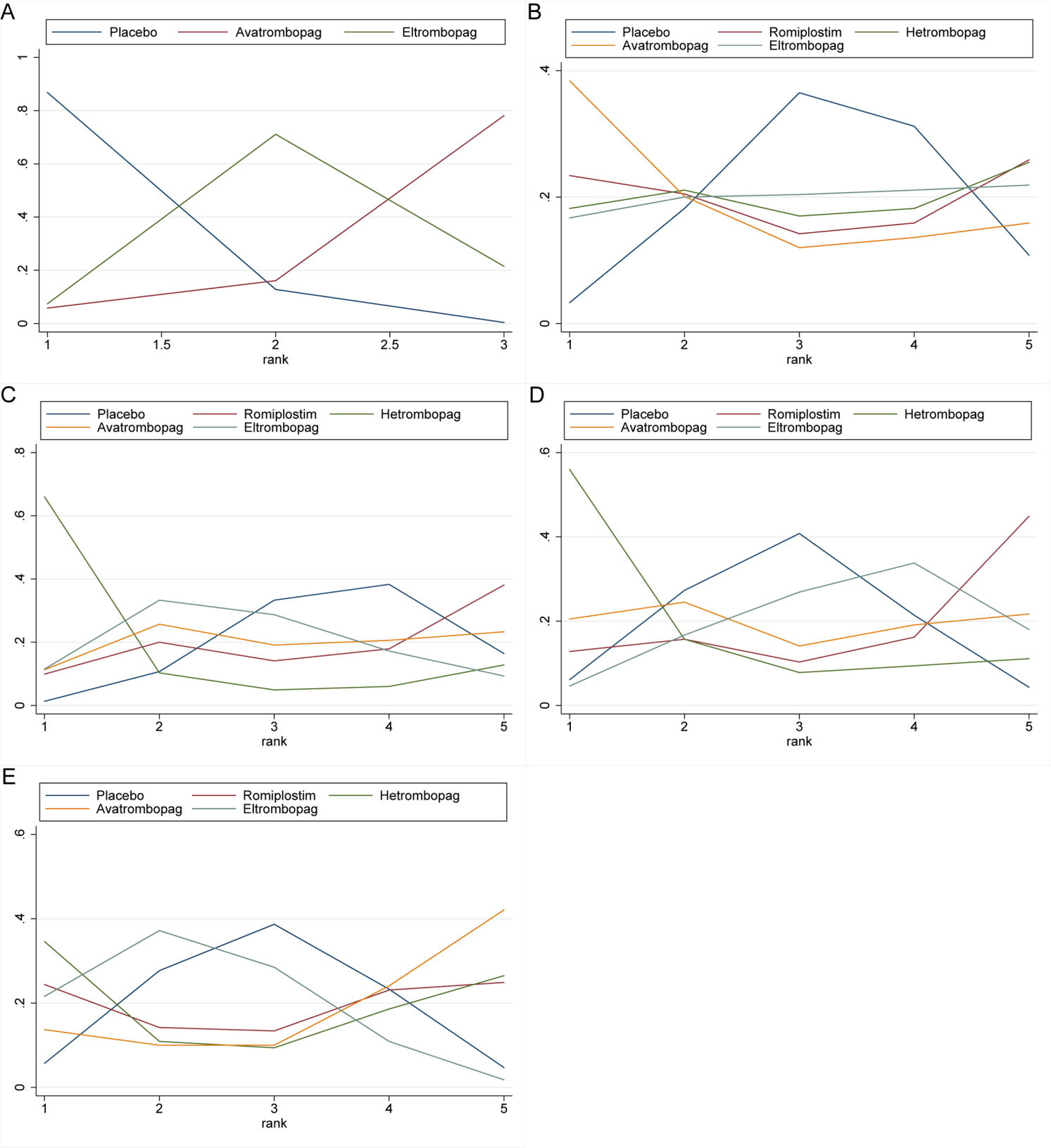


**Supplementary Figure 2.** Rank probability curves (Rankograms) for safety outcomes.

**(A)**platelet count >400×10^9^/L, **(B)**AEs, **(C)**serious AEs, **(D)**thrombosis, **(E)**mortality.

## 2.2 Supplementary Tables

**Supplementary Table 1.** League Table of chemotherapy dose reduction or delay due to thrombocytopenia.

| Hetrombopag |  |  |  |  |
| --- | --- | --- | --- | --- |
| 0.79 (0.44,1.42) | Eltrombopag |  |  |  |
| **0.45 (0.28,0.73)** | **0.57 (0.41,0.81)** | Placebo |  |  |
| **0.42 (0.18,0.96**) | 0.53 (0.24,1.14) | 0.92 (0.46,1.84) | Avatrombopag |  |
| 0.28 (0.07,1.19) | 0.36 (0.09,1.45) | 0.63 (0.16,2.42) | 0.68 (0.15,3.10) | Romiplostim |

# **Supplementary Table 2.** League Table of platelet transfusions.

| Hetrombopag |  |  |  |  |  |
| --- | --- | --- | --- | --- | --- |
| **0.29 (0.13,0.68)** | Placebo |  |  |  |  |
| 0.28 (0.06,1.17) | 0.95 (0.29,3.07) | Eltrombopag |  |  |  |
| 0.22 (0.05,1.03) | 0.77 (0.22,2.74) | 0.81 (0.14,4.59) | Avatrombopag |  |  |
| 0.20 (0.02,1.77) | 0.68 (0.09,5.13) | 0.72 (0.07,7.45) | 0.89 (0.08,9.64) | Romiplostim |  |
| **0.02 (0.00,0.86)** | 0.08 (0.00,2.68) | 0.08 (0.00,3.42) | 0.10 (0.00,4.34) | 0.11 (0.01,2.10) | Observation |

# **Supplementary Table 3.** League Table of grade 3 or 4 thrombocytopenia.

| Romiplostim |  |  |  |
| --- | --- | --- | --- |
| 0.96 (0.16,5.90) | Avatrombopag |  |  |
| 0.88 (0.20,3.98) | 0.92 (0.17,5.02) | Eltrombopag |  |
| 0.54 (0.17,1.72) | 0.56 (0.14,2.23) | 0.61 (0.23,1.63) | Placebo |

# **Supplementary Table 4.** League Table of bleeding events.

| Eltrombopag |  |  |  |
| --- | --- | --- | --- |
| 0.50 (0.07,3.79) | Avatrombopag |  |  |
| 0.37 (0.01,23.58) | 0.74 (0.01,57.21) | Hetrombopag |  |
| 0.41 (0.13,1.23) | 0.81 (0.15,4.42) | 1.10 (0.02,60.81) | Placebo |

# **Supplementary Table 5.** League Table of platelet count >400×10^9^/L.

| Placebo |  |  |
| --- | --- | --- |
| 0.66 (0.37,1.18) | Eltrombopag |  |
| 0.38 (0.12,1.24) | 0.58 (0.16,2.16) | Avatrombopag |

# **Supplementary Table 6.** League Table of AEs.

| Avatrombopag |  |  |  |  |
| --- | --- | --- | --- | --- |
| 0.97 (0.77,1.23) | Romiplostim |  |  |  |
| 0.97 (0.79,1.19) | 0.99 (0.81,1.22) | Eltrombopag |  |  |
| 0.97 (0.77,1.20) | 0.99 (0.80,1.23) | 1.00 (0.83,1.20) | Hetrombopag |  |
| 0.96 (0.81,1.14) | 0.99 (0.84,1.16) | 0.99 (0.88,1.12) | 1.00 (0.86,1.15) | Placebo |

# **Supplementary Table 7.** League Table of serious AEs.

| Hetrombopag |  |  |  |  |
| --- | --- | --- | --- | --- |
| 0.46 (0.04,4.88) | Eltrombopag |  |  |  |
| 0.40 (0.03,4.85) | 0.89 (0.28,2.85) | Avatrombopag |  |  |
| 0.35 (0.03,4.75) | 0.77 (0.19,3.04) | 0.87 (0.18,4.26) | Romiplostim |  |
| 0.37 (0.04,3.63) | 0.81 (0.43,1.53) | 0.91 (0.34,2.43) | 1.06 (0.30,3.71) | Placebo |

# **Supplementary Table 8.** League Table of thrombosis.

| Hetrombopag |  |  |  |  |
| --- | --- | --- | --- | --- |
| 0.37 (0.02,8.68) | Placebo |  |  |  |
| 0.38 (0.01,19.60) | 1.02 (0.10,10.97) | Avatrombopag |  |  |
| 0.30 (0.01,7.97) | 0.81 (0.32,2.02) | 0.79 (0.06,10.00) | Eltrombopag |  |
| 0.21 (0.00,15.04) | 0.56 (0.03,10.18) | 0.55 (0.01,23.15) | 0.70 (0.03,14.60) | Romiplostim |

# **Supplementary Table 9.** League Table of mortality.

| Eltrombopag |  |  |  |  |
| --- | --- | --- | --- | --- |
| 0.75 (0.01,38.24) | Hetrombopag |  |  |  |
| 0.83 (0.48,1.44) | 1.10 (0.02,53.98) | Placebo |  |  |
| 0.65 (0.03,13.46) | 0.87 (0.01,116.17) | 0.78 (0.04,15.42) | Romiplostim |  |
| 0.34 (0.02,7.21) | 0.45 (0.00,61.37) | 0.40 (0.02,8.27) | 0.52 (0.01,35.77) | Avatrombopag |

Interventions are reported in order of the outcome ranking according to SUCRAs. Comparisons should be read from left to right. The estimate (summary RR and 95% confidence intervals) is located at the intersection of the column-defining treatment and the row-defining treatment. RR value below 1 favours the column-defning treatment. To obtain RRs for comparisons in the opposing direction, reciprocals should be taken. Results were considered statistically significant if the 95%CI did not include null values (1 for RR). Significant results are in bold and underlined.

# Appendix 3 Grading the evidence for outcome of the network meta-analysis using CINeMA

1. CINeMA for the outcome “chemotherapy dose reduction or delay due to thrombocytopenia”


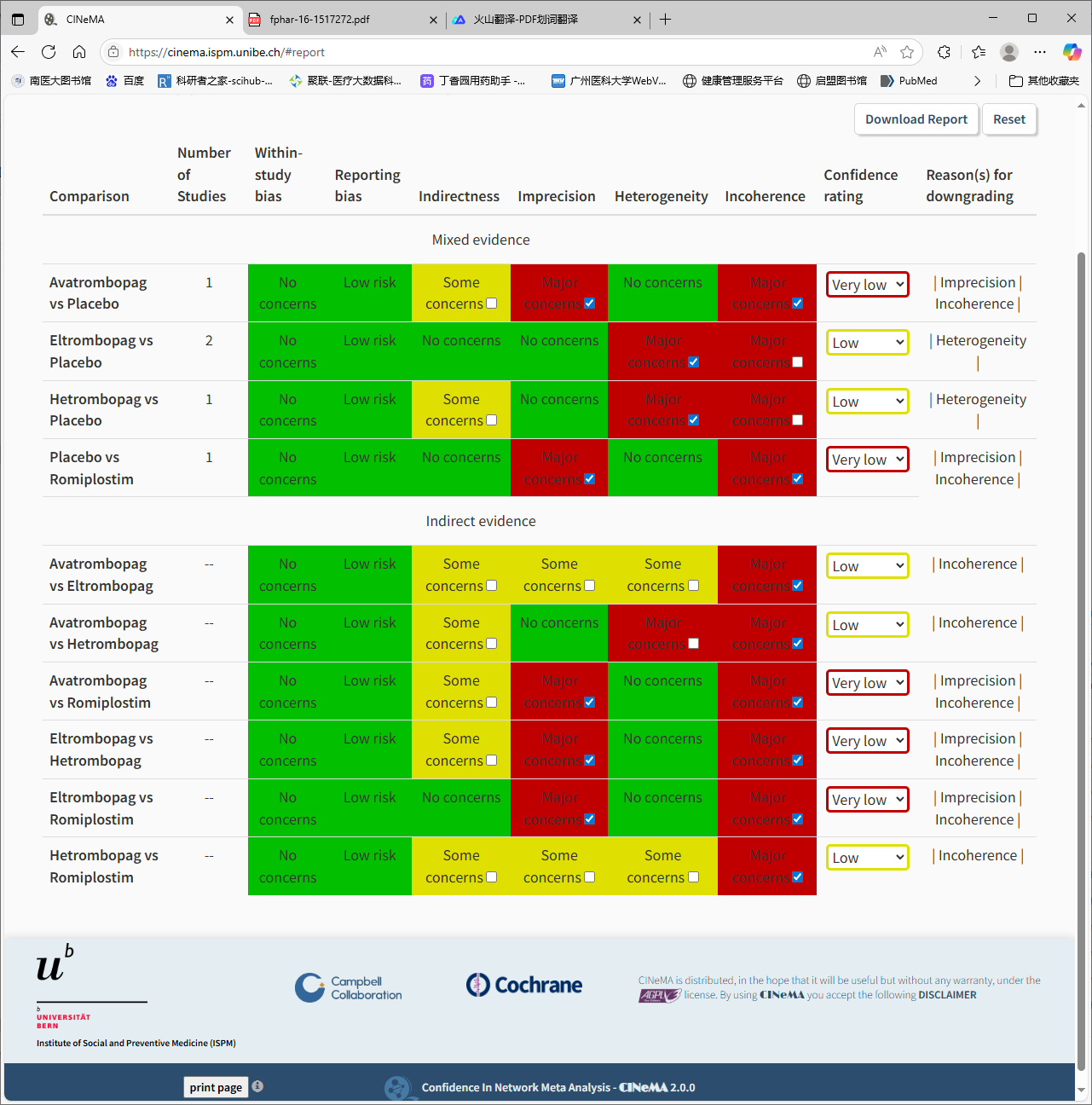


1. CINeMA for the outcome “platelet transfusions”


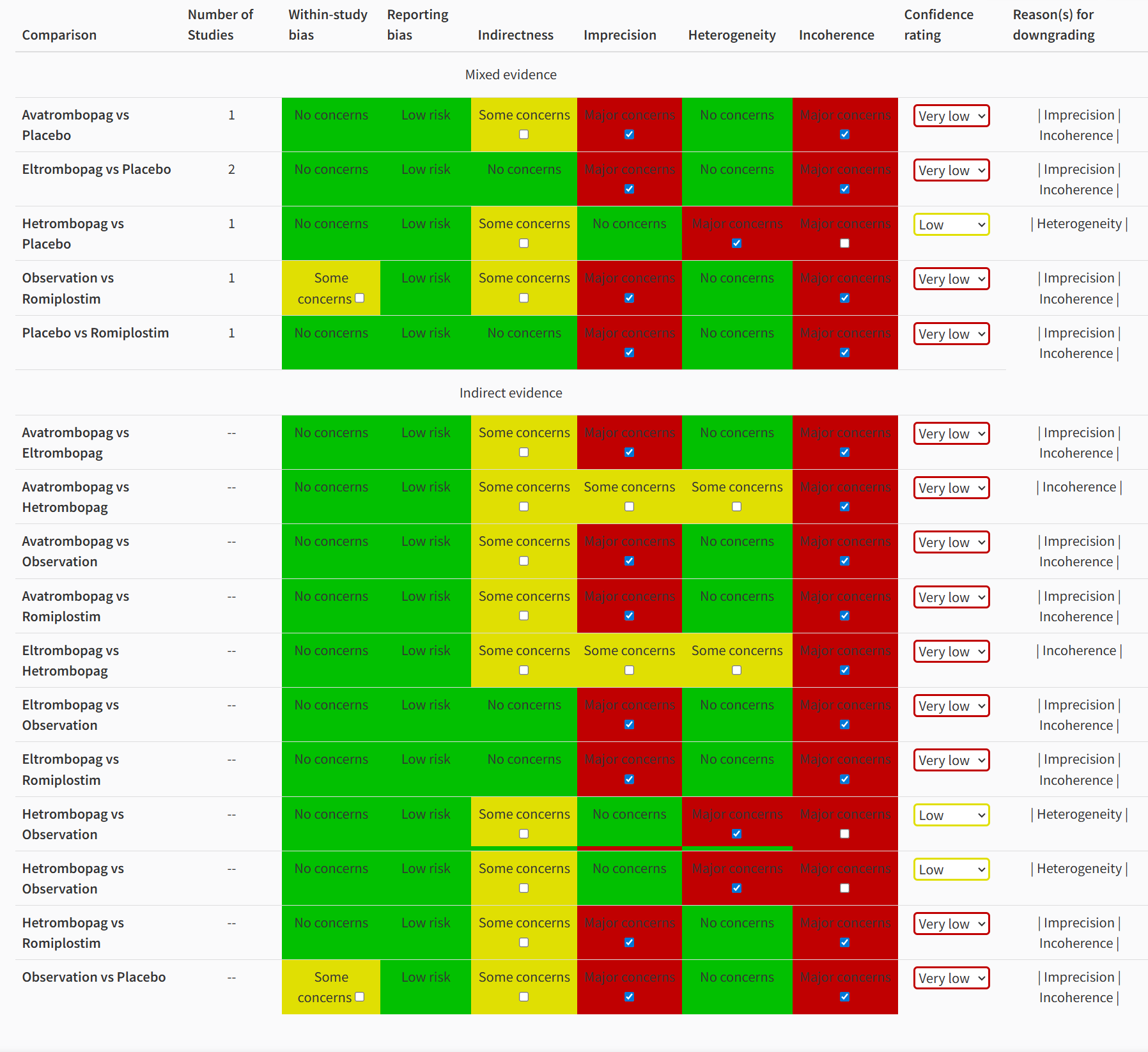


1. CINeMA for the outcome “incidence of grade 3 or 4 thrombocytopenia”


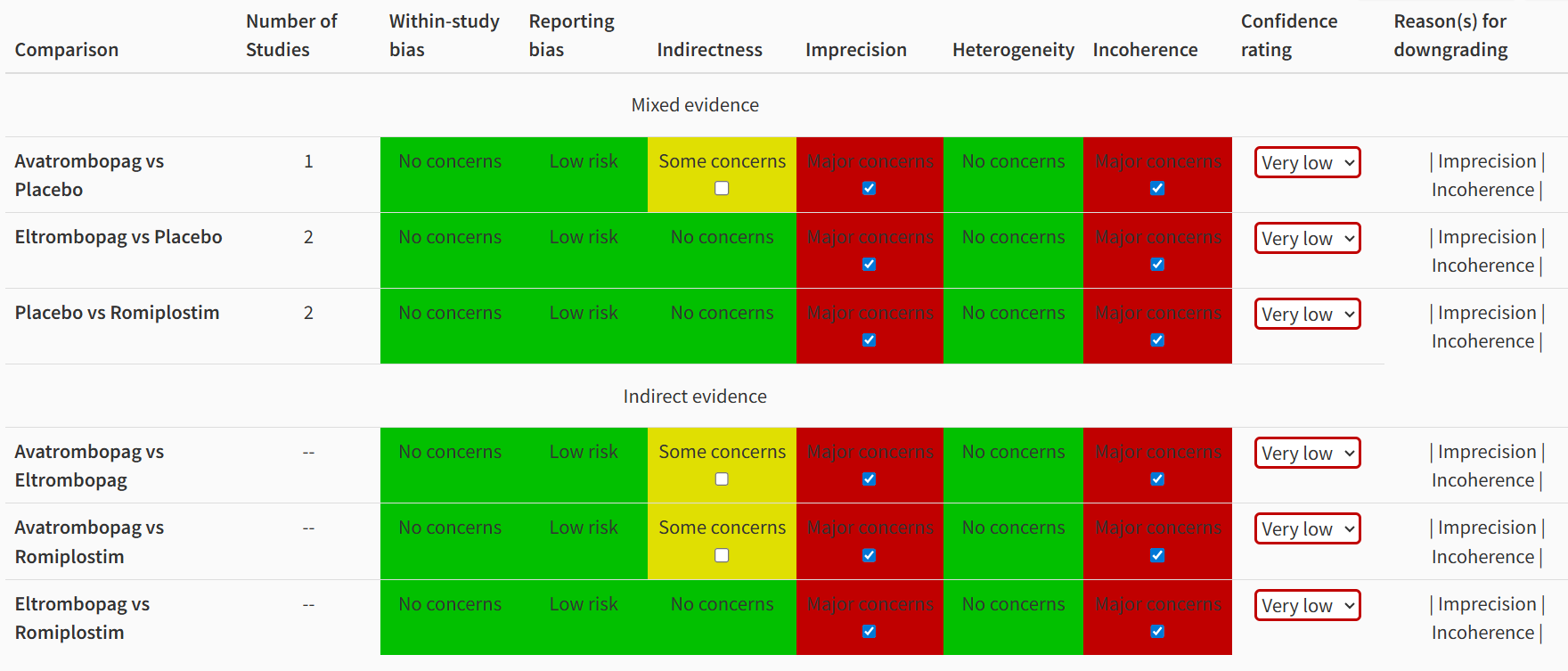


1. CINeMA for the outcome “bleeding events”


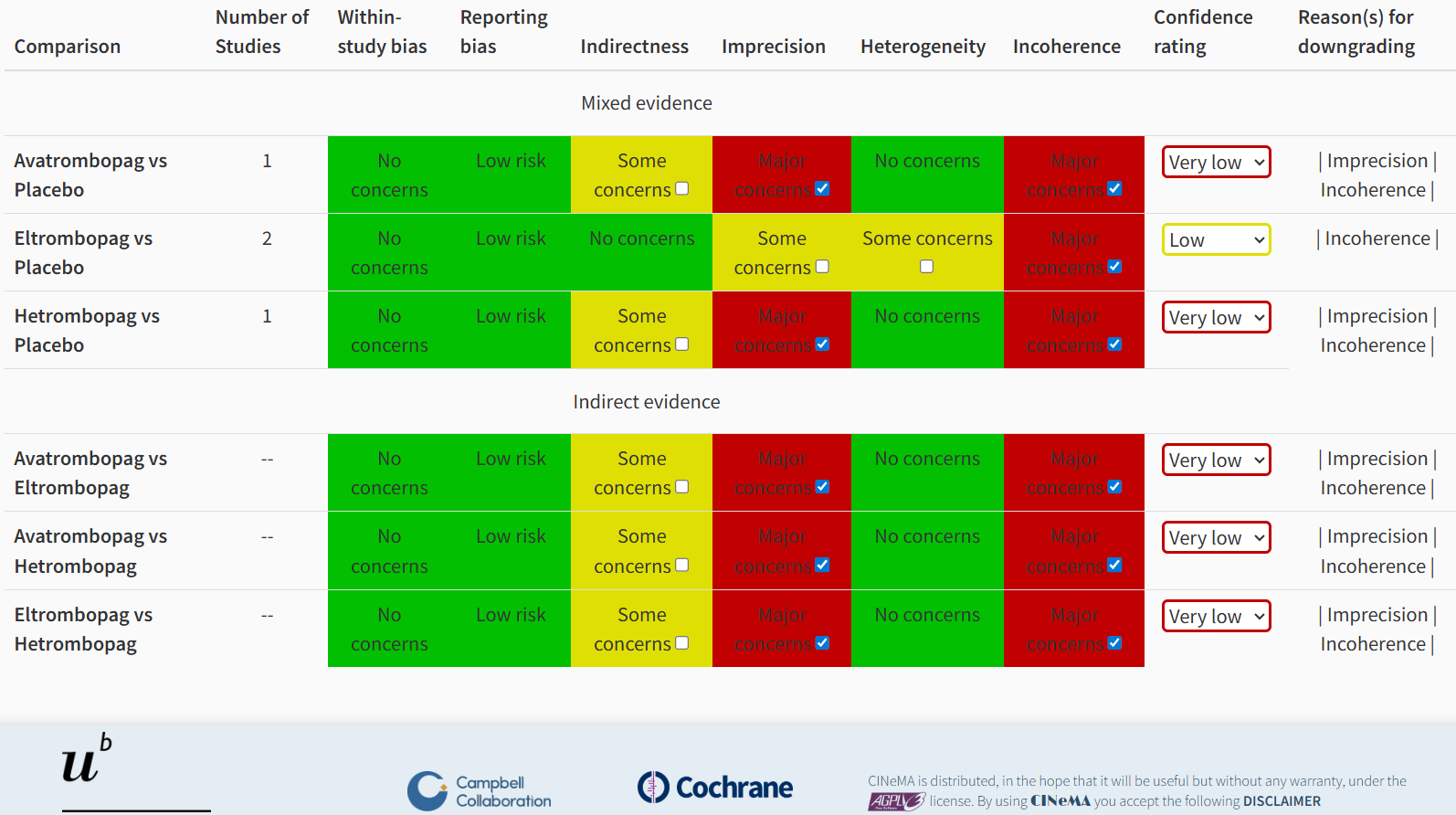


1. CINeMA for the outcome “platelet count >400×10^9^/L”


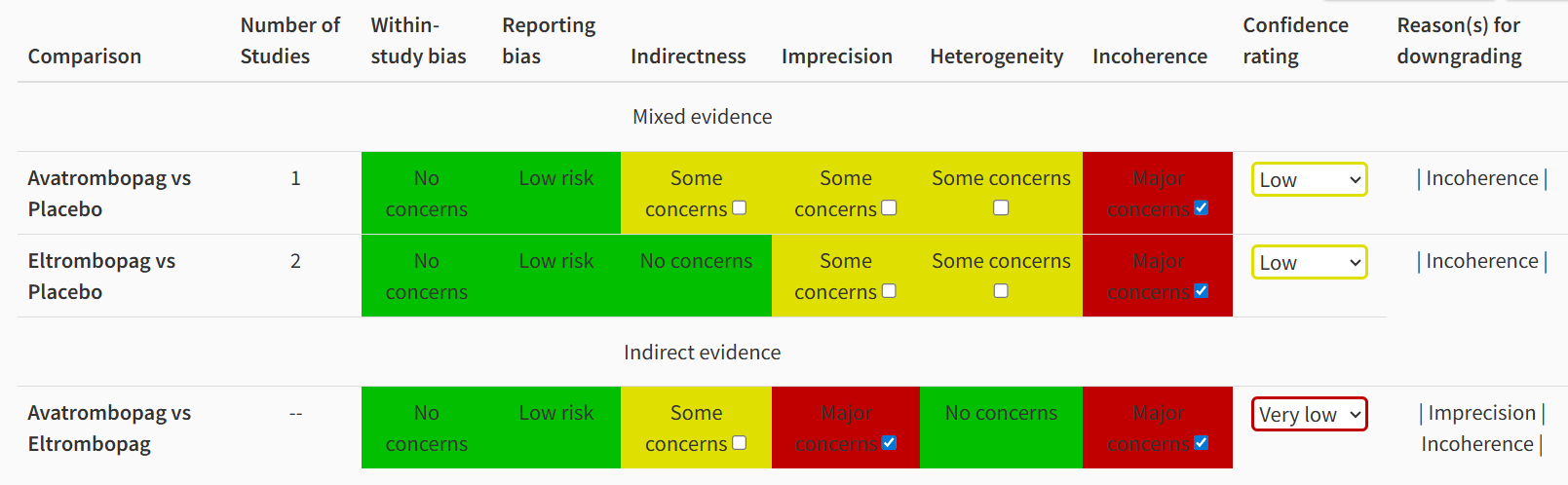


1. CINeMA for the outcome “AEs”


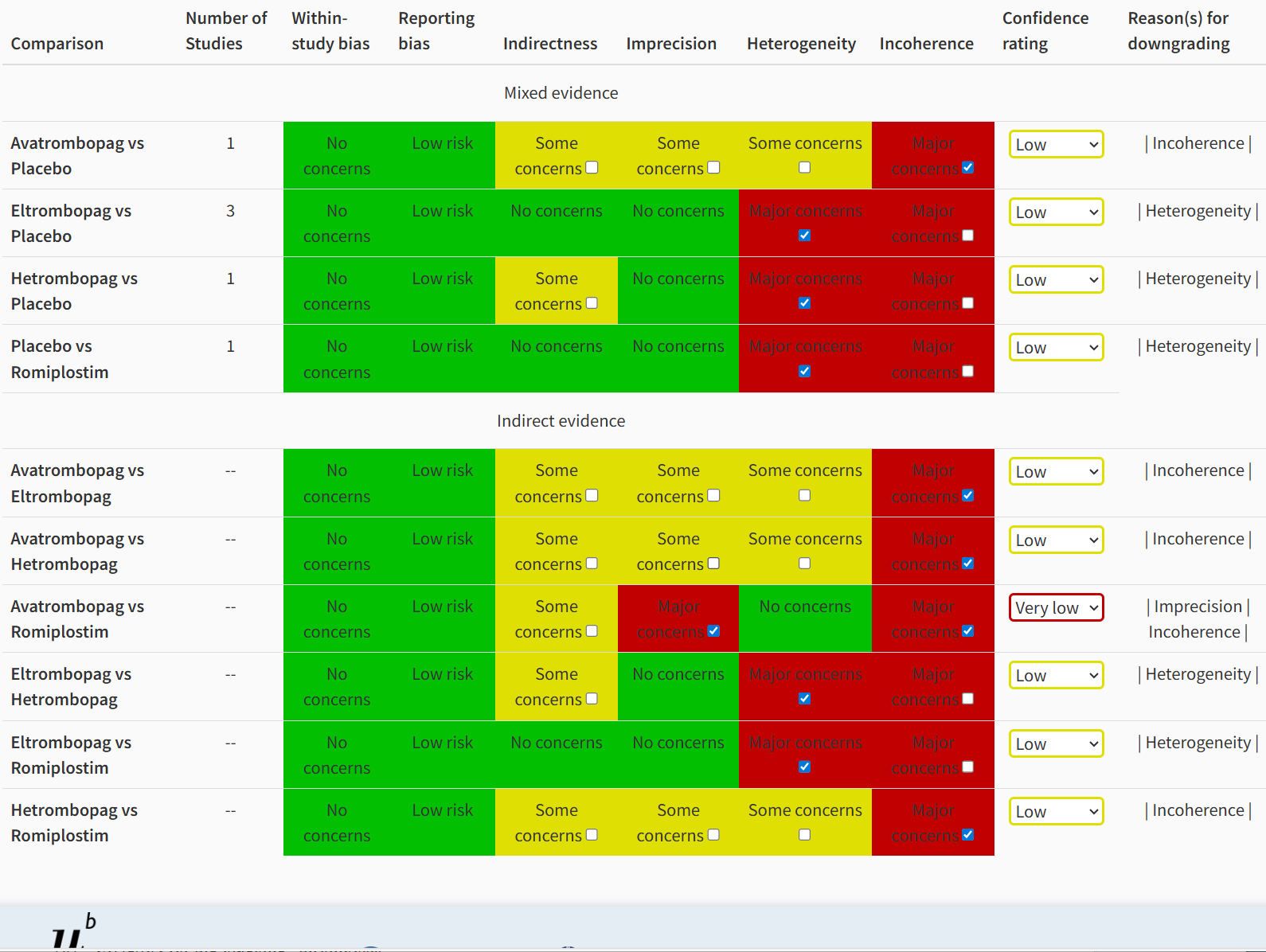


1. CINeMA for the outcome “serious AEs”


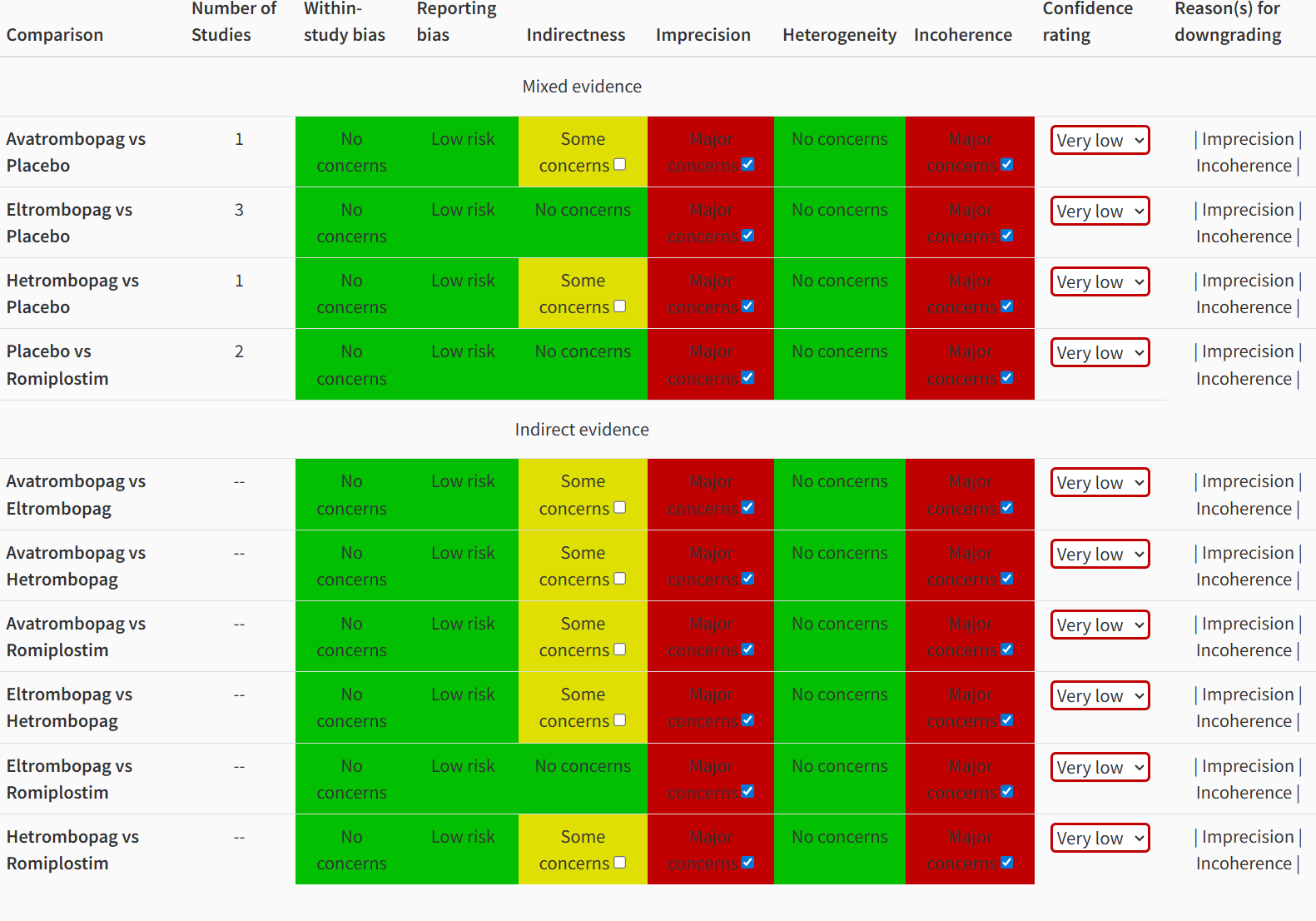


1. CINeMA for the outcome “thrombosis”


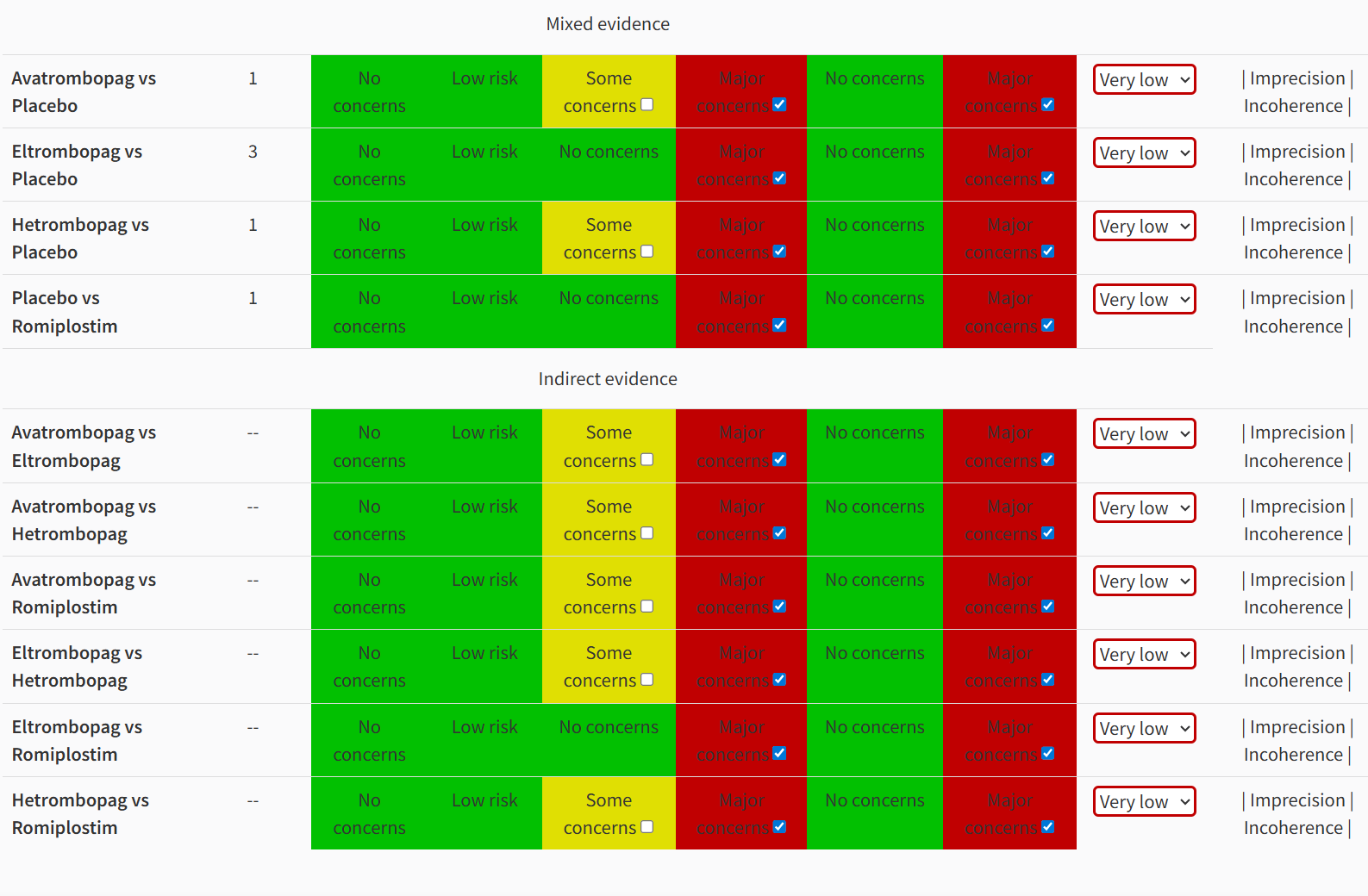


1. CINeMA for the outcome “mortality”


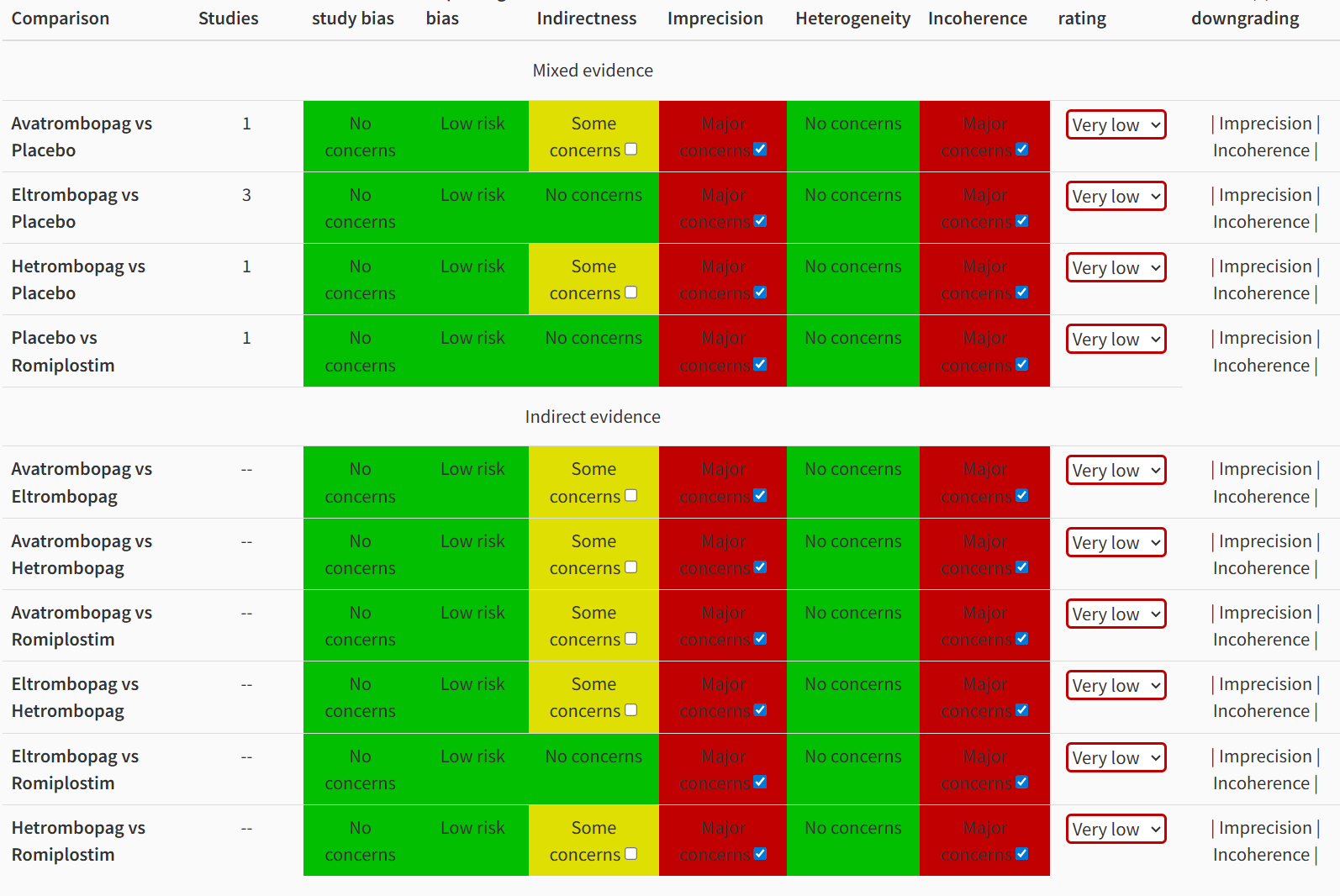

Supplement: Supplementary file 1 [file Supplementaryfile1.docx]
