# Supplementary figures and images for: The efficacy and safety of thrombopoietin receptor agonists in solid tumors with chemotherapy-induced thrombocytopenia: a systematic review and network meta-analysis of randomized controlled trials
Source: Front Pharmacol. 2025 Dec 1;16:1683857. doi: 10.3389/fphar.2025.1683857 (PMC12702962; doi:10.3389/fphar.2025.1683857)

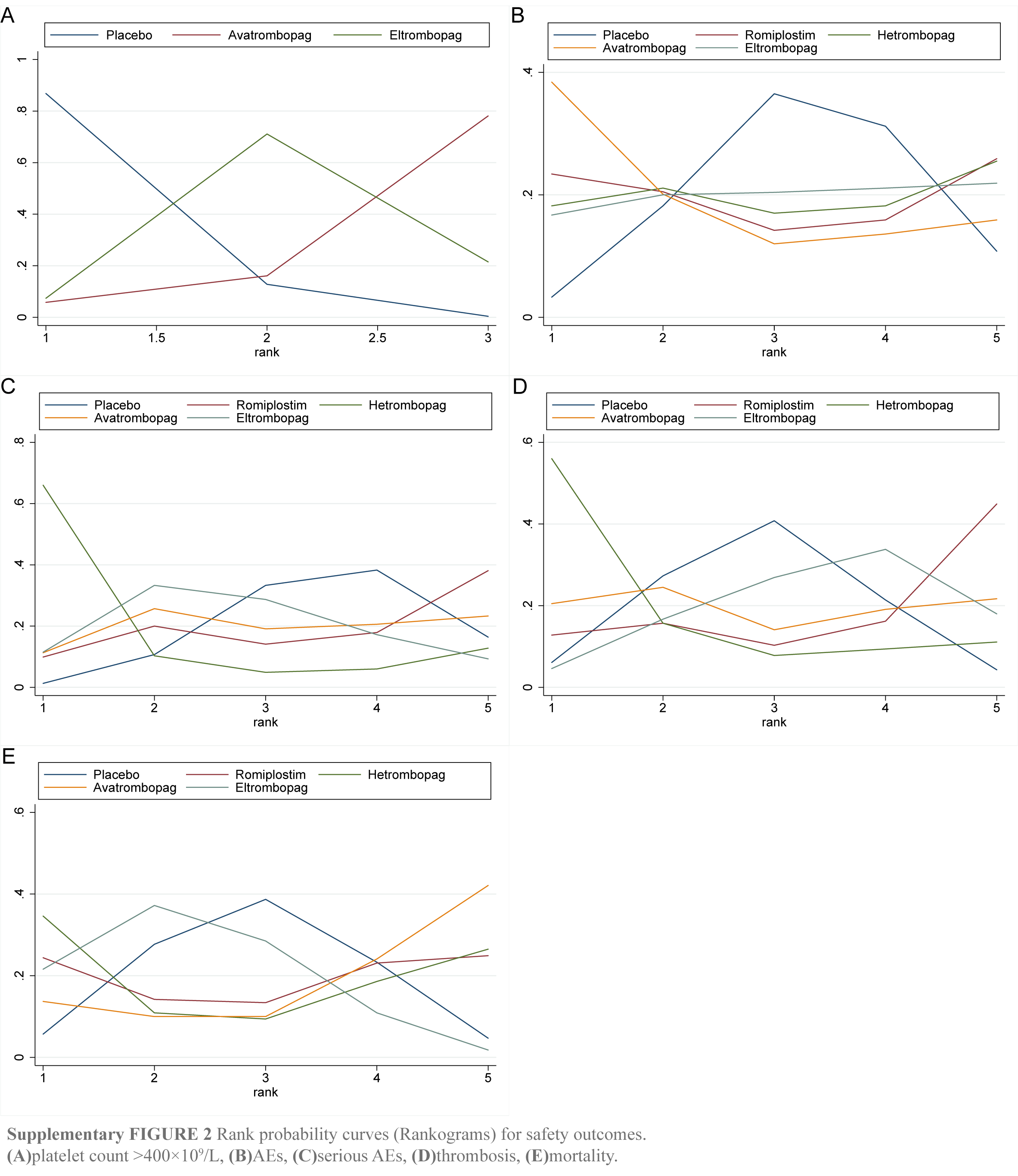

Supplement: Supplementary file 2 [file Image2.tif]

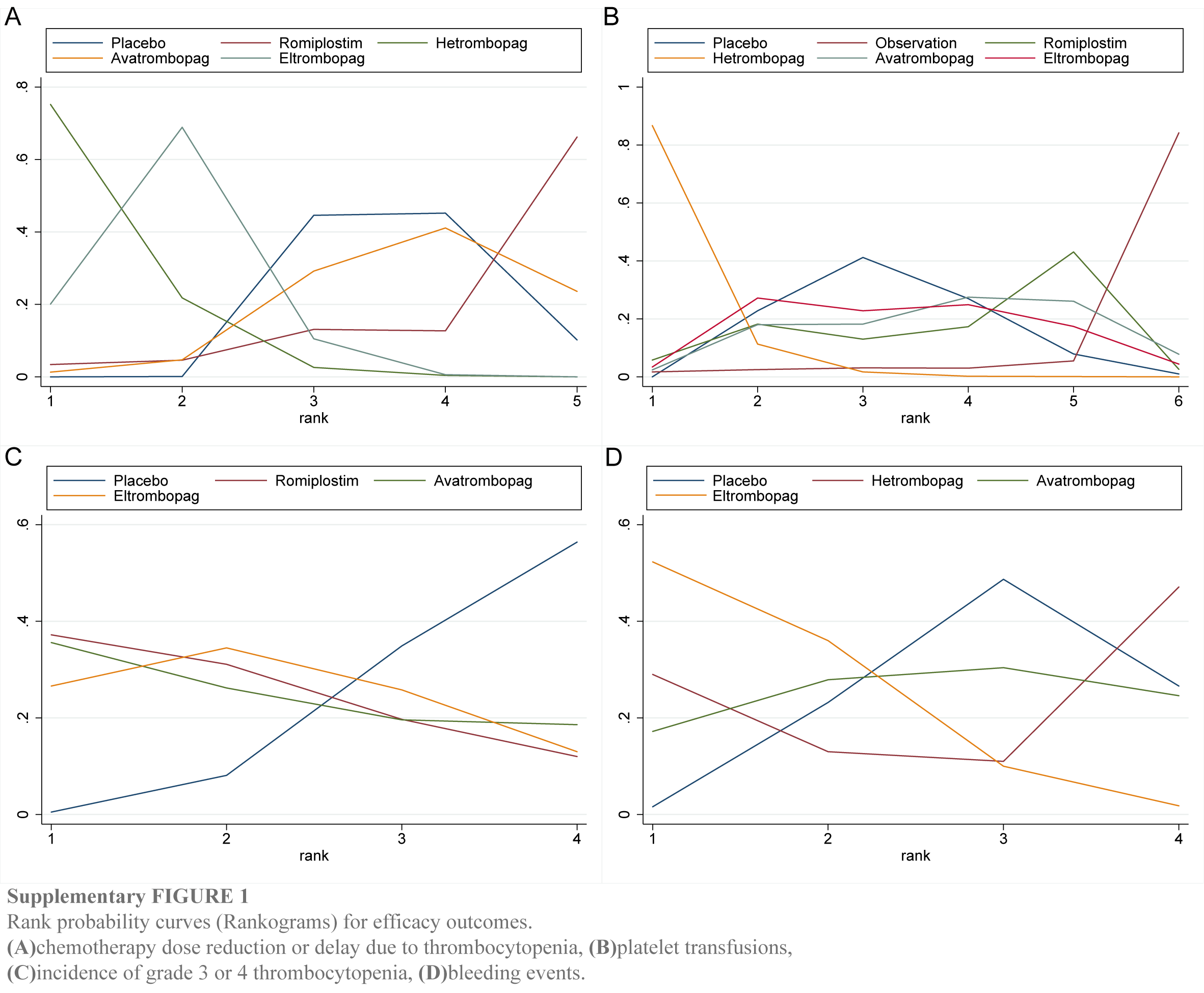

Supplement: Supplementary file 3 [file Image1.tif]
